# Non-Destructive Sensing and Intelligent Quality Prediction During Fruit Drying: From Quality Formation to Decision Support

**DOI:** 10.3390/foods15173122

**Published:** 2026-09-02

**Authors:** Kai Zhang, Qingqing Yuan, Tianrui Liu, Roujia Zhang, Lilang Li, Yu Wang, Siyao Liu, Chenguang Zhou

**Affiliations:** 1 China Light Industry Key Laboratory of Food Intelligent Detection & Processing, School of Food and Biological Engineering, Jiangsu University, Zhenjiang 212013, China; 15936056007@163.com (K.Z.); 19708897519@163.com (Q.Y.); 19709647735@163.com (T.L.); 1000005191@ujs.edu.cn (R.Z.); 2 State Key Laboratory of Discovery and Utilization of Functional Components in Traditional Chinese Medicine, Natural Products Research Center of Guizhou Province, Guizhou Medical University, Guiyang 550014, China; 18336131583@163.com (L.L.); wang_yu@gmc.edu.cn (Y.W.); 3 School of Pharmacy, Jiangsu University, Zhenjiang 212013, China

**Keywords:** fruit drying, quality formation, non-destructive sensing, intelligent prediction, process analytical technology, decision support

## Abstract

Fruit drying transforms a living, water-rich tissue into a stable food through coupled changes in moisture distribution, structure, color, nutrients, and aroma. Although drying technologies and non-destructive sensing have advanced rapidly, these fields have largely developed in parallel, leaving the relationship between quality formation and measurable process signals insufficiently resolved. Here, physical and chemical changes during drying are connected to the signals that can support quality prediction. Current evidence shows that moisture loss and surface appearance are the most tractable real-time targets. Texture, bioactive retention, and flavor remain less accessible because their signals depend more strongly on internal structure, reference chemistry, or sensory response. Optical, magnetic-resonance, thermal, volatile-sensing, and electrical approaches consequently provide complementary rather than interchangeable views of the product. Multimodal models improve prediction when the added signals resolve different aspects of drying, but redundant inputs can increase complexity without improving transferability. Progress toward intelligent fruit drying therefore depends on matching sensors to the evolving product state, validating models beyond individual batches and instruments, and linking predictions to practical process decisions. This process–quality perspective provides a basis for moving from retrospective quality assessment toward reliable monitoring and controlled drying.

## 1. Introduction

Fruit drying is a long-established preservation and value-added processing operation, but the quality of dried fruit is formed through more than water removal. During dehydration, fruit tissues undergo coupled heat and mass transfer, redistribution of free and immobilized water, cell-wall deformation, pore formation or collapse, enzymatic and non-enzymatic browning, oxidative reactions, nutrient transformation, and volatile-aroma remodeling [1,2,3]. For this reason, dried-fruit quality is better understood as a multi-attribute outcome involving color, texture, rehydration behavior, bioactive retention, flavor, storage stability, energy demand, and consumer acceptability, rather than as final moisture content or drying time alone [4].

Drying therefore follows changing transport and reaction regimes. Early in the process, surface evaporation and capillary flow dominate [5,6,7]. As surface water is depleted, intracellular diffusion, cell-wall resistance, solute concentration, and shrinkage increasingly limit transport; in late drying, low-moisture regions approach glass transition while browning, oxidation, and aroma loss continue. The effect of process intensification consequently depends on when and where energy is delivered [8,9,10]; shortening the early stage may be beneficial, whereas steep late-stage gradients can sacrifice structure or flavor. Reviews of hot-air, vacuum, freeze, infrared, microwave, refractance-window, electrohydrodynamic, hybrid, and pretreatment-assisted drying have documented these trade-offs mainly through kinetics, energy demand, and endpoint quality.

Non-destructive sensing offers a way to observe these transitions without repeatedly removing product from the dryer. Artificial intelligence (AI) has accelerated this development. Near-infrared (NIR) and visible–near-infrared (Vis–NIR) spectroscopy, hyperspectral imaging (HSI), low-field nuclear magnetic resonance (LF-NMR), magnetic resonance imaging (MRI), thermal imaging, machine vision, volatile-sensor arrays, and electrical sensing resolve different aspects of moisture, appearance, structure, and composition [11,12,13,14]. Machine-learning and data-fusion methods [15] then relate these measurements to moisture, quality attributes, or endpoints [16,17,18]. Much of the underlying sensing knowledge comes from fresh-fruit and broader agri-food inspection [19]; it establishes measurement principles and modeling constraints, but fruit-drying performance must still be judged in the changing optical, thermal, and structural environment of a dryer.

Drying and sensing research therefore meet at a specific unresolved problem: the former explains how process conditions shape endpoints, whereas the latter often predicts attributes without tracing the signal back to the transformations that produced it. Catalytic-infrared drying illustrates the process-centered tradition [20]. By contrast, fruit-quality sensing reviews commonly report predictive accuracy but give less attention to how moisture redistribution, structural remodeling, browning, nutrient loss, or volatile transformation gives rise to the measured signal [21]. Bridging the two requires a quality-formation framework that relates each sensing signal to the physical or chemical change that it can reasonably represent, distinguishes direct observation from calibration-dependent inference, and evaluates whether the resulting model remains useful across varieties, batches, instruments, and dryers.

Fruit drying is considered here as a continuous process in which quality-forming changes become measurable product states and, where current knowledge permits, process decisions. The analysis first establishes how moisture migration, structural remodeling, chemical reactions, raw-material characteristics, and processing conditions shape the quality trajectory. It then relates different sensors to complementary parts of that trajectory, examines their integration into quality prediction, and considers process analytical technology (PAT) in batch and continuous dryers. Table 1 situates this process–quality synthesis within the main strands of the previous review literature.

To support this synthesis, the Web of Science Core Collection was searched from database inception to 13 August 2026 using terms spanning fruit drying, non-destructive sensing, intelligent prediction, and quality formation. Of 4286 unique records, 275 were retained after document-type, language, and focused relevance assessment. The complete query and study-selection details are provided in Supplementary Table S1.

## 2. Quality Changes During Fruit Drying

### 2.1. Moisture Changes and Moisture Migration

Moisture migration is the primary physical event during fruit drying and the most direct determinant of drying rate [3]. As surface water evaporates, internal water must pass through cell walls, membranes, intercellular spaces and capillary pathways before reaching the surface. Consequently, water mobility and spatial distribution are as important as total moisture content [22,27]. LF-NMR and MRI distinguish free, immobilized and bound water populations and show that osmotic treatment, vacuum impregnation, freeze–thawing and drying temperature can alter their interconversion, thereby affecting effective moisture diffusivity, shrinkage, glass transition and rehydration [28,29]. This transport process is not quality-neutral: rapid surface evaporation may shorten drying time but intensify moisture gradients, case hardening, and non-uniform collapse, whereas moderate skin disruption or pore formation can reduce external resistance and improve drying uniformity. Citric-acid pretreatment during refractance-window drying of dragon fruit promoted pore formation and moisture transfer [30], while skin-disruption pretreatments in blueberries accelerated drying and altered shrinkage, rehydration and phenolic retention [31]. Mulberry maturity studies further confirm that water state links raw-material structure, drying behavior and final quality [32].

Case hardening develops when the surface loses mobility more rapidly than the wetter interior. In convectively dried papaya, shrinkage at 40 °C ceased near a wet-basis moisture content of 0.21 g g^−1^ as the glass-transition temperature approached the product temperature, whereas the matrix remained rubbery and continued shrinking at 70 °C [33,34]. Effective moisture diffusivity is similarly specific to the material and process model. Values for Bravo de Esmolfe apple ranged from 1.968 × 10^−10^ to 4.013 × 10^−10^ m^2^ s^−1^ in the first falling-rate period and from 0.9567 × 10^−10^ to 3.328 × 10^−10^ m^2^ s^−1^ in the second; Madeira banana values ranged from 1.572 × 10^−10^ to 2.627 × 10^−10^ m^2^ s^−1^ at 35–50 °C [35]. These measurements define transport behavior for the studied cultivars and geometries, but they do not provide universal thresholds for fruit drying.

Experimental studies clarify the distinction between total moisture loss and water-state redistribution. Total mass loss indicates how much water has been removed, whereas water-state redistribution determines how readily the remaining water can migrate and how strongly it interacts with cell walls, soluble solids, polysaccharides, and proteins [36]. Sun et al. related LF-NMR water populations to shrinkage during drying, showing that structural deformation can be interpreted through changes in water mobility rather than final moisture content alone [37]. Janowicz et al. demonstrated that osmotic pretreatment combined with vacuum impregnation modified water exchange in apples before drying and subsequently affected texture and rehydration [28]. Intermittent low-pressure superheated steam drying of mango also reduced shrinkage and improved rehydration while accelerating transport, indicating that mass-transfer enhancement and structural preservation can occur simultaneously when the process is appropriately controlled [38].

Pretreatment-assisted moisture migration therefore illustrates how physical and chemical changes become coupled during drying. Cold-plasma pretreatment can accelerate moisture diffusion and reduce energy demand through surface etching, microstructural modification, and changes in tissue permeability [7]. However, the response remains matrix-dependent [31,32]. Moderate surface modification may be advantageous for fruits with waxy cuticles or compact epidermal tissues, whereas the same treatment intensity may promote collapse, oxidation, or solute leakage in softer matrices. Moisture migration should therefore be interpreted as a tissue-specific transport process rather than as a universally positive response to process intensification.

### 2.2. Color Changes and Browning Reactions

Color is a sensitive indicator of thermal, enzymatic, and oxidative history during fruit drying. Changes in L*, a*, b*, total color difference and browning index may result from pigment degradation, polyphenol oxidase- or peroxidase-catalyzed browning, Maillard reactions, caramelization and the concentration of soluble solids. The dominant pathway depends on fruit composition: anthocyanins are sensitive to pH, oxygen and heat; carotenoids may degrade or isomerize; chlorophyll may convert to pheophytin; and tissue disruption can expose phenolic substrates to oxygen [39]. Color preservation is therefore not simply an appearance issue but a reflection of how heat load, enzyme activity and oxidation are controlled [40]. Drying-method comparisons further show that phytochemical stability and visual color do not always follow the same ranking, as observed for flavanone glycosides, polymethoxyflavones and antioxidant activity in orange peel [41].

Visible browning represents only part of the chemical damage associated with severe thermal exposure. Air-impingement drying of five jujube cultivars at 60 °C produced more N^ε^-carboxymethyllysine (CML), N^ε^-carboxyethyllysine (CEL), 5-hydroxymethylfurfural (HMF), and 3-deoxyglucosone than vacuum freeze drying [42]. CML and CEL indicate advanced glycation, whereas HMF may arise from 3-deoxyglucosone dehydration or sugar degradation. Evidence from commercial dried fruits and dried apricots also shows wide variation in HMF and reactive dicarbonyls with matrix, sulfuring, drying, and storage history [43,44]. These compounds can strengthen thermal-risk assessment within a defined product and process, although their concentrations cannot be converted into a common browning or safety threshold across fruits.

Color changes also have an optical–structural component. Pigment degradation and browning alter chromophore composition, whereas shrinkage, surface roughness, and porosity modify light scattering and perceived brightness. Intermittent low-pressure superheated steam drying of mango reduced total color difference and shrinkage relative to hot-air drying, suggesting that shorter thermal residence and better tissue preservation jointly improved visual quality [38]. During storage of dried coconut chips, color, crispness and peroxide value followed different kinetic patterns, and lipid oxidation was more closely associated with shelf-life loss than color alone [45]. Similarly, rapid vacuum-microwave drying of lemon slices required careful control of power and temperature to avoid local overheating and surface-quality deterioration [46]. These findings show that color should be interpreted together with structural and oxidative indicators rather than as an isolated endpoint.

The color response to pretreatment is particularly dependent on treatment intensity. Cold plasma, ultrasound, pulsed electric field, and advanced blanching can shorten drying time and improve moisture distribution, but they may also modify enzyme activity, oxygen access, and pigment chemistry when the processing window is not well controlled [47]. Moderate treatment may preserve color by reducing thermal exposure or inactivating browning enzymes [48], whereas excessive mechanical or oxidative stress may accelerate pigment degradation and surface browning. Color preservation therefore reflects a balance between transport enhancement and treatment-induced chemical stress.

### 2.3. Texture Changes and Tissue-Structure Remodeling

Texture formation in dried fruits is governed by the coupled evolution of water content, solid-matrix concentration, cell-wall integrity, pore structure, and glassy or rubbery state [22]. The porous-scaffold model proposed for freeze-dried fruits describes how polysaccharide networks, soluble sugars, and residual water jointly determine crispness and fracture behavior [49]. Other drying methods produce different combinations of open pores, compact collapse, and case hardening because their heating and pressure gradients differ. Accordingly, identical endpoint moisture contents may correspond to very different mechanical properties. Mango slices subjected to osmotic and microwave processing exhibited cut-dependent differences in shrinkage, image texture, and microstructure [50]; hybrid drying improved selected texture attributes of kiwifruit [51]; and freeze-drying/vacuum-microwave combinations generated distinct pore structures, crispness and rehydration behavior in pear slices [52]. These findings support interpreting texture through structure and water state rather than through hardness alone.

The initial tissue determines how the solid matrix develops as water is removed [32]. Cultivar, maturity, sugar composition, and cell-wall status influence both fresh firmness and the subsequent formation of pores, collapsed regions, and glassy domains. In plum, maturity-related differences in cell walls and water status were associated with drying behavior and phenolic profiles [53]. Cutting direction similarly affected the preservation of cellular organization in osmotically treated and microwave-dried mango. Texture formation therefore begins before drying and continues through shrinkage, pore development, and glass transition [54]. Comparisons among studies are most informative when maturity, tissue orientation, and composition are considered alongside the final mechanical response.

This structural perspective also explains why combined physical-field pretreatments do not necessarily produce linear improvements. Ultrasound can create cavitation, microjets and acoustic streaming that open transport pathways [55], whereas cold plasma generates reactive species predominantly near the surface and is limited by penetration depth [56]. Their combination may enhance mass transfer and matrix sensitization, but excessive mechanical–chemical coupling can enlarge pores, weaken cell walls, or promote oxidation. The optimum treatment is therefore not the greatest degree of disruption, but the formation of a connected pore network that facilitates water removal while preserving a mechanically stable solid matrix [49].

### 2.4. Changes in Bioactive Compounds

Bioactive compounds in fruits include ascorbic acid, phenolics, flavonoids, anthocyanins, carotenoids, and other phytochemicals that contribute to antioxidant capacity and functional value. Drying may reduce these compounds through thermal degradation, oxidation, enzymatic transformation, and prolonged oxygen exposure, but the response is not uniformly negative [57]. Tissue disruption can release bound compounds and increase extractability, while rapid or low-temperature drying may limit degradation. Interpretation should therefore distinguish true retention, release, transformation, and apparent concentration caused by water removal [41]. Vacuum freeze-drying of noni fruit preserved a more favorable active-metabolite profile than hot-air, microwave and far-infrared drying [58], whereas refractance-window drying of dragon fruit showed that suitable temperature and citric-acid pretreatment could improve phenolic retention and antioxidant activity [30]. Ultrasound-assisted osmotic pretreatment of longan likewise produced beneficial effects only within moderate combinations of intensity, duration and temperature [59].

Phytochemical classes do not respond uniformly to drying [60]. Reviews of fruit-slice drying generally associate vacuum, hybrid, and freeze-drying processes with better retention of phenolics, flavonoids, carotenoids, anthocyanins, and ascorbic acid than prolonged high-temperature drying, but also emphasize strong matrix dependence [61]. The noni comparison above accordingly revealed method-specific metabolite profiles rather than a uniform change in total bioactivity. In orange peel, polymethoxyflavones were more stable across drying methods than the major flavanone glycosides [41]. Compound-specific or profile-level evidence is therefore more informative than a single total-content or antioxidant assay when different biochemical pathways respond independently.

Bioactive-compound retention also affects product functionality and valorization potential. Drying can reduce seasonal losses and convert fruit or by-product streams into higher-value ingredients, but the value of the resulting product depends on whether heat-sensitive and oxidation-sensitive compounds are preserved or transformed in a desirable way. Low-temperature, vacuum, freeze, hybrid, and moderate nonthermal pretreatments may offer advantages for selected compounds, yet outcomes remain dependent on fruit matrix, maturity, oxygen exposure and assay design. Nutrient retention is therefore both a compositional result and a determinant of whether dried fruit can serve as a nutrient-dense snack or a higher-value functional ingredient [1].

### 2.5. Flavor Changes and Volatile-Compound Losses

Flavor changes during drying arise from simultaneous volatilization, thermal degradation, oxidation, Maillard and Strecker reactions, and the concentration or de novo formation of selected aroma-active compounds [62]. Because individual volatiles differ in odor threshold and interact within a matrix composed of numerous esters, aldehydes, alcohols, ketones, terpenes, sulfur compounds, and furans, total volatile abundance does not directly represent sensory quality. Consumer acceptance depends on perceived aroma intensity, odor balance, and texture–flavor interactions; gas chromatography–mass spectrometry (GC–MS) or electronic-nose signals therefore require aroma-activity or sensory context when flavor is used as a quality endpoint. Drying can reconstruct the odor space rather than simply weaken fresh-fruit aroma [63]. In sapodilla, the dominant perception shifted from minty, fatty-green, and woody notes toward citrusy, balsamic, and sweet notes after drying [64]. During raspberry fruit-foam processing, seed separation and final freeze-drying caused substantial losses of major odorants, whereas microwave-assisted freeze-drying improved the retention of selected aroma compounds [65]. Electronic-nose and GC–MS analysis of jujube further showed that volatile patterns continue to evolve during both drying and storage [66]. Flavor assessment must therefore distinguish desirable aroma formation from oxidation, cooked notes, and over-processing and relate compound-level changes to sensory relevance.

Odor activity values (OAVs) relate compound concentration to the odor threshold in the relevant matrix and thus offer a closer approximation of potential aroma contribution than concentration alone. Compounds with an OAV of at least one are commonly prioritized, whereas gas chromatography–olfactometry (GC–O) identifies which eluting compounds are perceived; their combined use has revealed characteristic aroma compounds in dried peach slices [67,68]. Odor thresholds and mixture interactions nevertheless vary by matrix. Summed OAVs therefore cannot be interpreted as a direct measure of sensory intensity.

Cold plasma (CP) research provides an additional example of treatment-induced flavor remodeling. CP can promote rearrangement, hydrolysis, hydrogenation, ring opening and ring closure of volatile compounds, producing changes in alcohols, aldehydes, carbonyls, furans, terpenes and other aroma-related groups [26]. Voltage, frequency, feed gas, flow rate and treatment duration may either reduce undesirable notes or intensify oxidation-derived volatiles. Flavor preservation during drying therefore requires control not only of total volatile loss, but also of which compounds are retained, transformed or newly generated and whether these changes are perceptible in sensory evaluation.

### 2.6. Coupling and Trade-Offs Among Multiple Quality Attributes

Fruit quality develops through shared transport and reaction pathways, so changes in one attribute often carry consequences for another. Faster water removal can shorten thermal exposure and preserve selected nutrients, yet steep moisture or temperature gradients may intensify case hardening, local overheating, and volatile loss. Color preservation may likewise coincide with undesirable texture or aroma because pigments, pore structure, and volatile compounds respond differently to water activity, oxygen, composition, and temperature. Within this process–quality context, adequate endpoint moisture or water activity, limited thermal damage, oxidative stability, and avoidance of mold associated with under-drying act as quality-stability constraints [1,39]. Single-index evaluation can therefore obscure the balance that determines product acceptability [69]. Physical-field and hybrid methods are beneficial when faster transport is not offset by greater structural or chemical damage [5].

Multi-attribute studies reveal how these balances shift with product and process. In dried coconut chips, color, crispness, and lipid oxidation followed different storage kinetics, and the attribute governing shelf-life loss was not the most visually apparent one [45,70]. Electrohydrodynamic drying combined favorable energy and exergy performance with phenolic and color retention, although drying rate and scale-up remained limiting [71]. AI-based optimization studies similarly show that moisture stability, color, texture, nutrients, aroma, energy, and throughput may respond differently to the same operating variable [17]. Process selection therefore depends on explicit priorities and constraints rather than on the expectation that every quality attribute can be maximized simultaneously.

**Figure 1:**
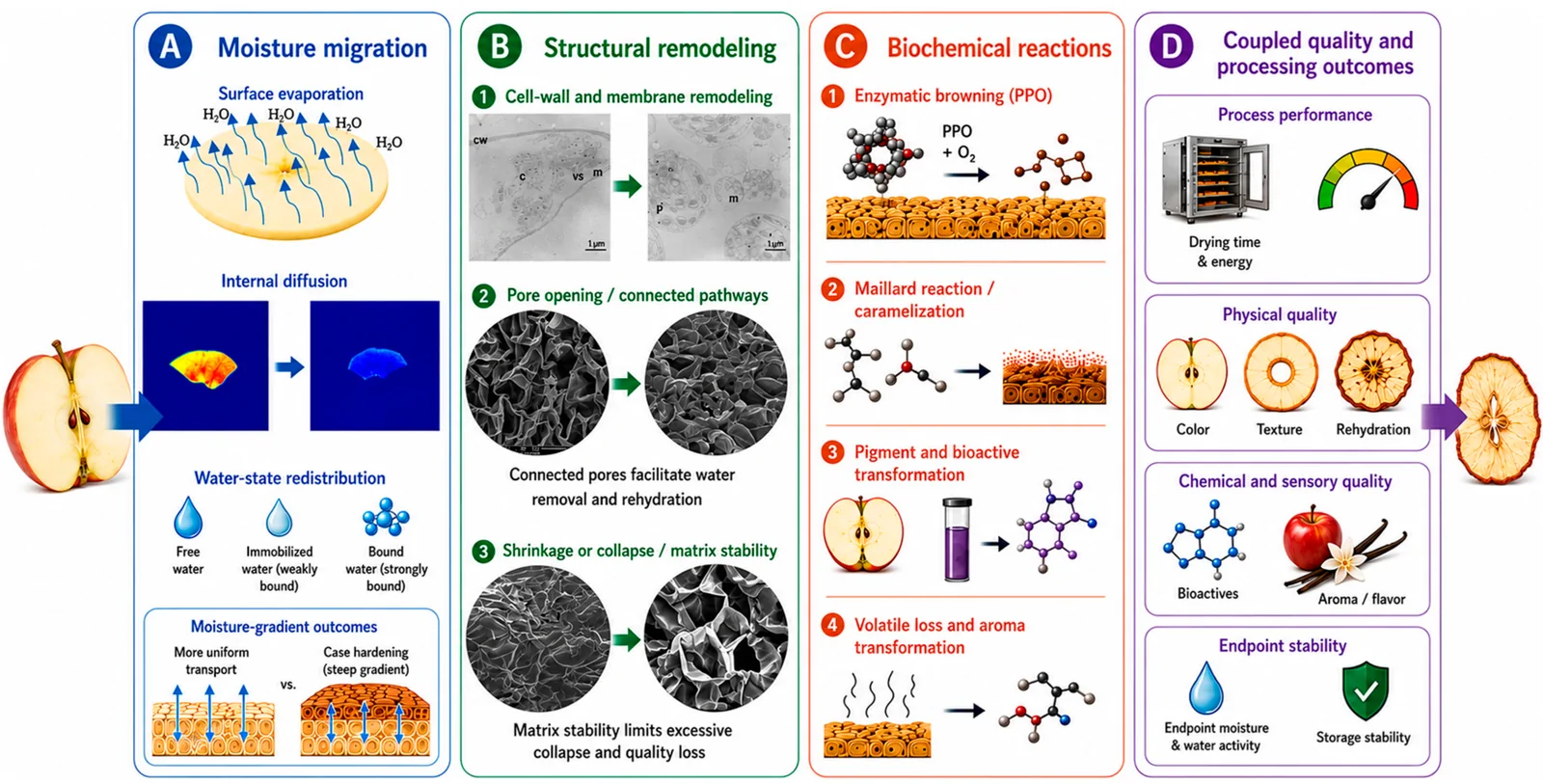
Schematic diagram of the drying-induced quality-formation pathway in fruit, linking moisture migration, structural remodeling, biochemical reactions, and coupled process and quality outcomes. The magnetic resonance imaging (MRI) water-distribution images and scanning electron microscopy (SEM) micrographs were adapted from Ref. [73] with permission. The transmission electron microscopy (TEM) micrographs were adapted from Figure 5E4 and Figure 5G4 of Ref. [74] under the terms of the Creative Commons Attribution License (CC BY 4.0). Cropping, relabeling, and layout adjustments were performed for consistency. Abbreviations in the TEM micrographs: cw, cell wall; c, chloroplast; vs, vesicle; m, mitochondrion; p, plastoglobuli.

Sustainability-oriented drying reviews extend this multi-attribute framework from product quality to process- and system-level performance. Drying contributes to food security, waste valorization, clean-energy use and circular food systems, but laboratory-scale quality advantages may not translate into sustainable production when equipment cost, energy demand, technical training or operational complexity are excessive [8]. Tray drying of white-fleshed peach illustrates how a conventional process can valorize a perishable fruit stream, although a single product study does not by itself establish system-level sustainability [72]. Conversely, a lower-cost method may be valuable in decentralized processing when combined with reliable endpoint sensing. The most informative evaluation therefore integrates product quality with process efficiency, resource use, scalability and socioeconomic context. Figure 1 summarizes the integrated pathway from moisture migration and structural remodeling to biochemical reactions and coupled quality outcomes.

## 3. Differences in Quality Changes Among Fruit Matrices and Drying Conditions

### 3.1. Differences Among Fruit Species and Cultivars

Fruit species and cultivars differ in moisture content, soluble solids, acidity, pigment composition, cell-wall structure, peel thickness, wax layers, seed distribution and aroma precursors. These variables determine both the initial state of the tissue and its trajectory during drying. High-sugar fruits are more susceptible to stickiness, glass-transition shifts and late-stage collapse; anthocyanin-rich fruits are more sensitive to pH, oxygen and heat; and peel-protected fruits may exhibit strong external mass-transfer resistance. Concrete studies illustrate this matrix dependence. Density-based grading of mulberry linked maturity-related changes in water-binding capacity and microstructure with drying time, volatile composition, color, texture and sensory quality [32]. Plum maturity was similarly associated with cell-wall composition, water status, drying characteristics and phenolic profiles [53], while peach cultivar and sugar composition influenced osmotic mass transfer and health-related attributes [75]. Cultivar and maturity should therefore be treated as explanatory variables rather than experimental noise [76].

The same matrix differences alter non-destructive signals and model transfer [77]. Reviews of mango, citrus, avocado, and pear assessment identify soluble solids, dry matter, firmness, acidity, oil content, surface defects, and anatomy as cultivar- and maturity-dependent sources of variation [11,78]. Together with evidence from peach and dried wolfberry [79], these findings show that composition and morphology can change both drying behavior and the signal recorded by a sensor. A model may therefore respond differently across cultivars even when temperature and slice thickness are nominally identical. Maturity, firmness, dry matter, surface barriers, geometry, and pretreatment history provide essential context for separating matrix effects from apparent differences between dryers or algorithms [28,80].

### 3.2. Effects of Tissue Part and Sample Geometry

Sample geometry determines diffusion-path length, exposed surface area, internal moisture gradients and the degree of structural deformation [28,81]. Slice thickness, diameter, cut direction, tissue orientation and peel retention influence whether drying is controlled mainly by internal diffusion, skin resistance or surface evaporation. In blueberries, the waxy cuticle slows moisture removal and contributes to bursting, surface sugar adhesion and bioactive losses, whereas laser perforation, ultrasound and freeze–thaw pretreatments improve drying rate and rehydration by modifying this barrier [31]. Mango slices with oval, longitudinal and transversal cuts also differ in shrinkage, color, image texture and microstructure during osmotic and microwave processing [50]. Peel and by-product tissues, including orange or pomegranate peel, contain dense structural barriers and high phenolic or pigment concentrations and may therefore respond differently from fleshy tissues to permeability-enhancing pretreatments [82]. Geometry and tissue part are thus mechanistic determinants of both mass transfer and quality development [75].

Geometry also controls how sensor signals are interpreted [83]. Thick slices may retain strong internal moisture gradients while appearing dry at the surface, whereas thin slices dry rapidly but are more exposed to oxidation, edge effects and surface hardening. Although these reviews address internal visualization and tuber quality rather than fruit drying directly, they provide transferable methodological insight into how sensor configuration, signal processing, and the spatial distribution of water, starch, defects, and compositional heterogeneity affect non-destructive grading [84,85]. For fruit-drying applications, this implication should be translated to fruit-specific matrices, where soluble solids, cuticle properties, cellular porosity and tissue orientation replace starch-rich tuber structure as the main sources of spatial heterogeneity. Consequently, thickness, orientation, and tissue part should be reported and, where possible, incorporated into calibration models rather than treated as minor preparation details.

### 3.3. Differences Among Drying Methods

Drying methods create distinct quality trajectories because they differ in energy delivery, pressure, humidity, temperature uniformity, and internal heating [86]. Hot-air drying is economical and robust but may require prolonged exposure, increasing shrinkage, browning, and nutrient loss [72]. Freeze drying usually preserves pore structure, color, and rehydration at the expense of time and energy [52]. Microwave and vacuum-microwave systems accelerate internal heating but can create hotspots or collapse, while infrared, refractance-window, and low-pressure steam systems produce their own surface and matrix effects [30,61]. Experiments illustrate rather than eliminate these trade-offs: low-pressure superheated steam shortened mango drying while limiting color change and shrinkage [38]; vacuum freeze drying preserved a favorable noni metabolite profile [58]; hybrid drying improved selected kiwifruit attributes [51]; and electrohydrodynamic drying reduced energy demand but retained scale-up constraints [71]. The appropriate method consequently depends on the intended product and the attribute most vulnerable during processing.

The link between transport mechanism and product use becomes especially important when rapid energy delivery is involved. In lemon slices, vacuum-microwave drying was advantageous only when power and temperature were controlled sufficiently to limit hotspots; lower-temperature operation protected thermolabile compounds but required longer treatment or greater equipment demand [46]. Citrus drying shows a similar dependence on power, temperature, and endpoint control [87]. Across technologies, drying time and energy must therefore be considered together with structure, nutrients, aroma, scale-up, and compatibility with process monitoring [8,88]. No single ranking remains valid when the desired product changes.

### 3.4. Effects of Pretreatments on Quality Changes

Pretreatments modify drying quality by changing cell-membrane permeability, peel resistance, pore formation, solute distribution, enzyme activity, and antioxidant protection [56,89]. Ultrasound, pulsed electric field, cold plasma, freeze–thawing and microwave-assisted treatments can intensify mass transfer, whereas osmotic dehydration and impregnation additionally modify solids content and mechanical properties. Meta-analytical and review evidence indicates that ultrasound and other nonthermal approaches can shorten drying time, reduce hardness and improve selected color or functional attributes, but the response depends on treatment mode, power, duration and material structure [47,90]. A sequential pulsed-electric-field and ultrasound-assisted convective-drying study on orange peel further showed that pretreatment changes quality parameters as well as transport behavior [82]. Pretreatment should therefore be viewed as a controlled intervention that alters both transport and quality-forming reactions, not simply as an auxiliary step for accelerating dehydration.

Pretreatment effects usually follow a processing window rather than a monotonic response to intensity. In ultrasound-assisted osmotic treatment of longan, suitable combinations of sucrose, citric acid, calcium chloride, ultrasound, and drying temperature preserved structure and antioxidant-related quality, whereas stronger treatment accelerated losses of vitamin C and polyphenols [59]. Vacuum impregnation also altered water exchange, texture, and rehydration in apple before drying [28]. Increased permeability is therefore useful only while solute leakage, structural weakening, and chemical degradation remain limited [91].

**Figure 2:**
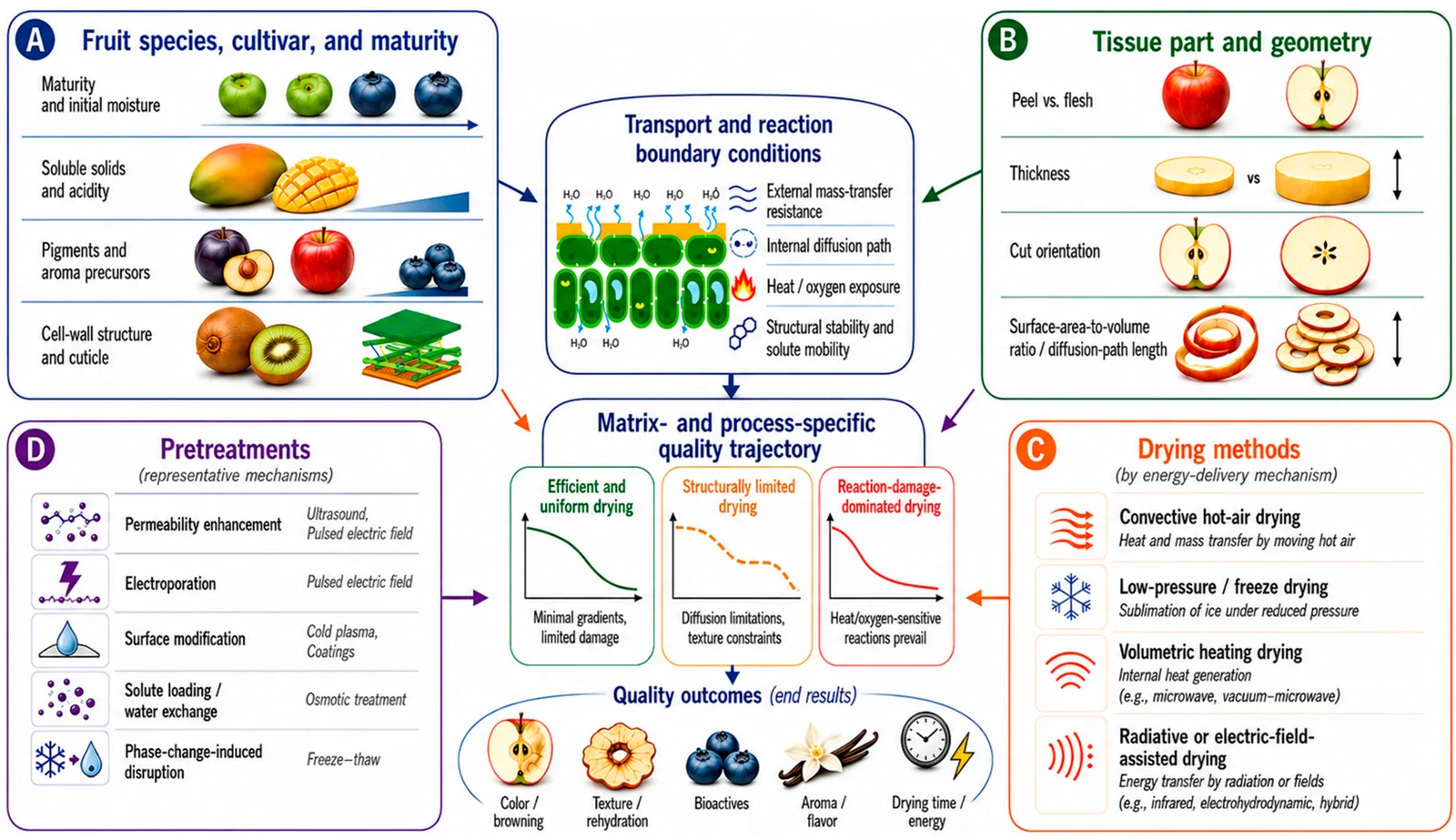
Schematic diagram of matrix- and process-specific quality trajectories during fruit drying, highlighting the effects of fruit species, cultivar, maturity, tissue geometry, drying method, and pretreatment on transport–reaction boundary conditions and final quality outcomes. The cell-wall structure panel was adapted from Ref. [7] with permission. Cropping, relabeling, and layout adjustments were performed for consistency.

Recent pretreatment studies converge on a balance between transport enhancement and quality protection [92]. Cold plasma can promote moisture diffusion through surface etching and microstructural change [7], whereas ultrasound creates cavitation, microjets, and acoustic streaming. Ultrasound-generated pathways may increase the access of plasma-derived reactive species, coupling mechanical and chemical effects [56]. This interaction can support mass transfer and enzyme control, but excessive exposure may enlarge pores, accelerate oxidation, or weaken the matrix. The useful treatment range therefore changes with the tissue rather than increasing uniformly with applied intensity. Figure 2 summarizes how raw-material and process conditions shape these quality trajectories.

## 4. Non-Destructive Sensing of Quality Changes During Fruit Drying

The matrix and process effects described above generate spatially and temporally different trajectories of moisture, temperature, structure, and composition. Each sensing method reveals only part of this evolving state. Optical images describe the surface, spectra respond to water and chemical absorptions within a limited sampling depth, magnetic resonance resolves water mobility and distribution, and process sensors describe the drying environment. Multimodal sensing becomes useful when these perspectives resolve different uncertainties in the same product rather than when instruments are simply accumulated [75,83,85].

### 4.1. Non-Destructive Sensing of Moisture Changes

Moisture content, moisture ratio, and endpoint status are the most suitable targets for online monitoring because they change continuously during drying and directly affect process termination and product stability [93]. Inadequate moisture removal compromises storage stability, whereas unnecessary overdrying increases energy demand and can intensify quality loss [39]. NIR spectroscopy and hyperspectral imaging are established optical tools because water produces strong O-H-related absorption and drying alters spatial scattering behavior [23,24,25,94]. Dielectric sensing and mass-based measurements provide complementary bulk or process-scale information [95]. However, these methods do not measure the same moisture scale. Mass sensors provide a bulk dehydration trend; NIR mainly reflects averaged absorption within a limited optical penetration depth; HSI adds surface-near spatial heterogeneity; dielectric or impedance signals respond to bulk water, ionic mobility, and temperature; and LF-NMR/MRI reveals water mobility and spatial distribution rather than only total water [37]. Thus, moisture is the most realistic online target when the measurement scale is explicitly matched to the control objective.

Apple-drying studies conducted between 900 and 1700 nm repeatedly identified moisture-sensitive responses near 950–970, 1190, 1450, and 1680 nm; a portable NIR study selected 1359, 1517, and 1594 nm [23,96]. These bands reflect the instruments, temperature, scattering, penetration depth, and tissue structures examined in those studies. Their recurrence nevertheless suggests a practical development path in which full spectra identify stable regions and reduced-band systems are subsequently tested on independent drying batches.

The performance of a moisture model depends as much on signal origin and sample heterogeneity as on the reported coefficient of determination [93]. Apple studies show that NIR spectra can replace destructive moisture assays under controlled conditions, while Vis-NIR measurements have tracked moisture ratio during jujube drying [97]. HSI becomes more informative when dehydration is spatially non-uniform or surface and core states diverge, whereas LF-NMR is particularly valuable for relating water populations to shrinkage and rehydration [37]. High accuracy within one fruit, geometry, or dryer may therefore reflect a narrow calibration range or a strong association with elapsed time rather than a generally transferable water response.

These differences create a continuum from routine process monitoring to mechanistic diagnosis [85]. Mass, temperature, humidity, dielectric, and selected-band optical sensors are comparatively amenable to online use. NIR balances speed with sensitivity to water and composition, while HSI adds spatial information at greater cost and computational demand. LF-NMR and MRI are more suited to resolving water-state transitions during model development [95,98,99]. Combining these measurements is useful when their sampling scales and information content are complementary. Near the endpoint, reliable prediction also requires validation across batches and dryer conditions and a warning when the observed product falls outside the calibration domain.

### 4.2. Non-Destructive Sensing of Color and Appearance Changes

Color and appearance are well suited to online observation because their changes occur at the product surface. RGB imaging can continuously quantify L*, a*, b*, hue, area, shrinkage, wrinkling, and visible defects at comparatively low cost [100]. These features reveal browning, deformation, and drying progress, but they do not establish nutrient retention, internal texture, or flavor. HSI and three-dimensional imaging add spectral and geometric information, while thermal imaging can reveal uneven heat loading before visible browning is pronounced [101,102].

The meaning of an image feature depends on the decision it is used to support [103]. Color and morphology have been related to moisture ratio and appearance during kiwifruit drying [104], and spectral-spatial measurements have followed quality changes in convectively dried apple [83]. Surface features therefore provide useful indicators of process progression. Their relationship with the internal product state weakens, however, when similar colors conceal different moisture gradients, pore structures, or aroma losses. Models based on appearance are most reliable when their target is specified as surface grading, browning warning, or endpoint assistance rather than undifferentiated product quality.

For routine monitoring, RGB imaging can follow color, area, and shrinkage, while thermal imaging identifies local heating and selected spectral bands resolve subtler pigment or moisture changes [105,106]. Full HSI provides richer diagnostic information but also increases cost, data volume, and sensitivity to illumination and sample presentation. Surface surveillance is therefore closer to industrial application than inference of internal quality, which still benefits from moisture-, structure-, or composition-sensitive measurements [12,13].

### 4.3. Indirect Sensing of Texture and Tissue-Structure Changes

Texture and tissue-structure changes are difficult to sense directly because they emerge from cell-wall integrity, pore architecture, residual water, glass transition and fracture mechanics [49]. Non-destructive texture prediction relies on indirect descriptors such as shrinkage, image texture, surface roughness, spectral scattering, LF-NMR water populations, ultrasound propagation and 3D geometry [107]. The key issue is that the same moisture content may correspond to different textures: one product may retain an open porous network, whereas another collapses or hardens [108]. For this reason, texture is not an ideal single-sensor online target; it is better treated as a structural outcome inferred from multiple signals and calibrated against destructive or sensory reference tests.

These examples indicate that the transferability of texture models depends on whether image or spectral features reflect the same structural mechanisms across products and drying methods [109]. Image texture and microstructure explain quality differences between mango cut types and osmotic/microwave treatments [50], and hyperspectral plus 3D point-cloud approaches separate shrinkage, deformation and physicochemical changes that two-dimensional images may miss [101]. However, a correlation between an image feature and hardness is not necessarily transferable. It holds when both are driven by surface wrinkling, but fails when fracture behavior is dominated by internal pores, sugar-glass transitions or cell-wall damage. Texture models need raw-material descriptors, geometry, moisture-state information and clearly defined reference labels such as hardness, crispness, chewiness or rehydration capacity.

Texture-related signals are strongest when surface deformation, water mobility, and internal structure are interpreted together. HSI and three-dimensional features can track shrinkage, collapse, or hardening during drying, although evidence from broader NIR-HSI research does not establish the same prediction performance for every dried fruit [108]. LF-NMR links structural change with water state, while ultrasound and mechanical measurements help determine whether a predicted texture reflects density gradients, interfaces, or fracture behavior [110,111]. Acoustic velocity, attenuation, and impedance are sensitive to internal heterogeneity, but their relation to texture still requires standardized reference conditions [112]. A model based on these complementary descriptors is less dependent on a single hardness value that may represent only one fracture mode.

### 4.4. Spectral Sensing of Bioactive Compounds

Spectral sensing of bioactive compounds is attractive because conventional assays for phenolics, flavonoids, vitamin C, anthocyanins and antioxidant capacity are destructive and time-consuming [61]. However, these compounds are among the least direct targets for online spectral prediction. NIR and HSI capture O-H-, C-H- and N-H-related absorptions together with scattering changes caused by the matrix [113], but total phenolics or antioxidant capacity often reflect correlations with water, color, browning or tissue density rather than a unique compound-specific signal [13,98]. This risk increases during drying because water removal changes baselines, concentration effects and light scattering. Bioactive models are best interpreted as calibrated inference rather than direct chemical measurement [114].

Spectral models of bioactive quality are most useful for screening trends and identifying quality risk [115,116]. Explainable artificial intelligence (XAI)-guided HSI can reduce wavelength redundancy and visualize spatial variation [117], while selected-band short-wave infrared (SWIR) systems offer a less complex route to moisture- and carbohydrate-related information [118]. Their chemical meaning depends on rigorous reference assays, independent validation, and physically plausible wavelength interpretation [119]. At present, bioactive prediction complements moisture and appearance monitoring rather than providing an independent online assay.

### 4.5. Electronic-Nose and Volatile-Fingerprint Sensing of Flavor Changes

Electronic noses (e-noses) and gas-sensor arrays follow shifts in the headspace during drying, including changes associated with oxidation and off-flavor formation [120]. Their output is a composite response to volatiles, humidity, and background gases rather than a direct identification of individual compounds [121]. This makes them sensitive to the direction and timing of aroma change, while chemical and sensory analyses remain necessary to explain what that change means. Studies of sapodilla, raspberry foam, and jujube show that dehydration reorganizes the volatile profile, while sensory importance depends on compound-specific odor thresholds and matrix-dependent mixture interactions rather than concentration alone (Section 2.5) [64,65,66]. In a dryer, e-nose signals are therefore most valuable as temporal fingerprints anchored by periodic chromatographic or sensory reference measurements.

Rapid volatile fingerprints become more informative when they are connected to chemical identity and sensory relevance. Multimodal colorimetric sensing during chili-pepper drying illustrates this approach in another food matrix [122]. Electronic noses, tongues, and eyes provide holistic descriptors, whereas GC–MS, GC–O, and sensory analysis distinguish desirable aroma development from oxidative or cooked notes. This distinction is particularly important after cold-plasma or other oxidative pretreatments because alcohols, aldehydes, ketones, furans, and terpenes can shift in favorable or undesirable directions [26]. Models that combine volatile fingerprints with process conditions and sensory or chromatographic labels therefore carry more meaning than models based on total peak area.

In industrial settings, electronic noses can provide an early warning of changes in odor space during late drying or storage. Periodic GC–MS or sensory assessment supplies compound-level and perceptual interpretation; dried Gardenia storage provides an example of volatile changes associated with packaging [123]. Moisture, temperature, and image features may add useful context when aroma loss coincides with oxidation, browning, or local overheating. Transfer among fruits and dryers, however, remains sensitive to humidity, drift, and background gases.

### 4.6. Complementary Sensing Across the Drying Process

The sensing literature reveals a consistent division between product states that are readily observable and quality attributes that require inference [124]. Moisture progression, mass loss, surface color, shrinkage, and product temperature have relatively direct physical expressions. Texture, rehydration, bioactive retention, and flavor perception emerge from several structural, chemical, or sensory processes and therefore depend more strongly on calibrated relationships. The coupled effects of impregnation and ultrasound on freeze-dried apple quality illustrate why one surface signal cannot represent the resulting structure and composition [125]. Machine vision, environmental and mass sensors, and selected-band NIR or multispectral measurements are consequently suited to repeated process observation. Electrical responses can add moisture-state information when geometry, contact, and temperature are controlled [95], whereas HSI, LF-NMR/MRI, ultrasound, GC-MS, and texture tests remain especially valuable for calibration and diagnosis.

Instrument capability is inseparable from measurement geometry. Optical methods are constrained by penetration, scattering, illumination, sample shape, and surface temperature [126]. HSI adds spatial–spectral detail but increases acquisition and processing demands; electronic noses respond to humidity and background gases as well as volatiles; dielectric and impedance measurements depend on ionic mobility, contact, geometry, and temperature; and LF-NMR/MRI resolves water state but is difficult to embed in a dryer [93]. Imaging and spectroscopy for toxigenic fungi and aflatoxins provide a broader methodological example of how heterogeneous distributions and matrix interference constrain sensor interpretation [127]. These limitations determine whether a signal represents the surface, a sampled bulk volume, or a proxy calibrated against another measurement. The most useful sensor is therefore the one that resolves the physical uncertainty relevant to the intended process decision.

**Figure 3:**
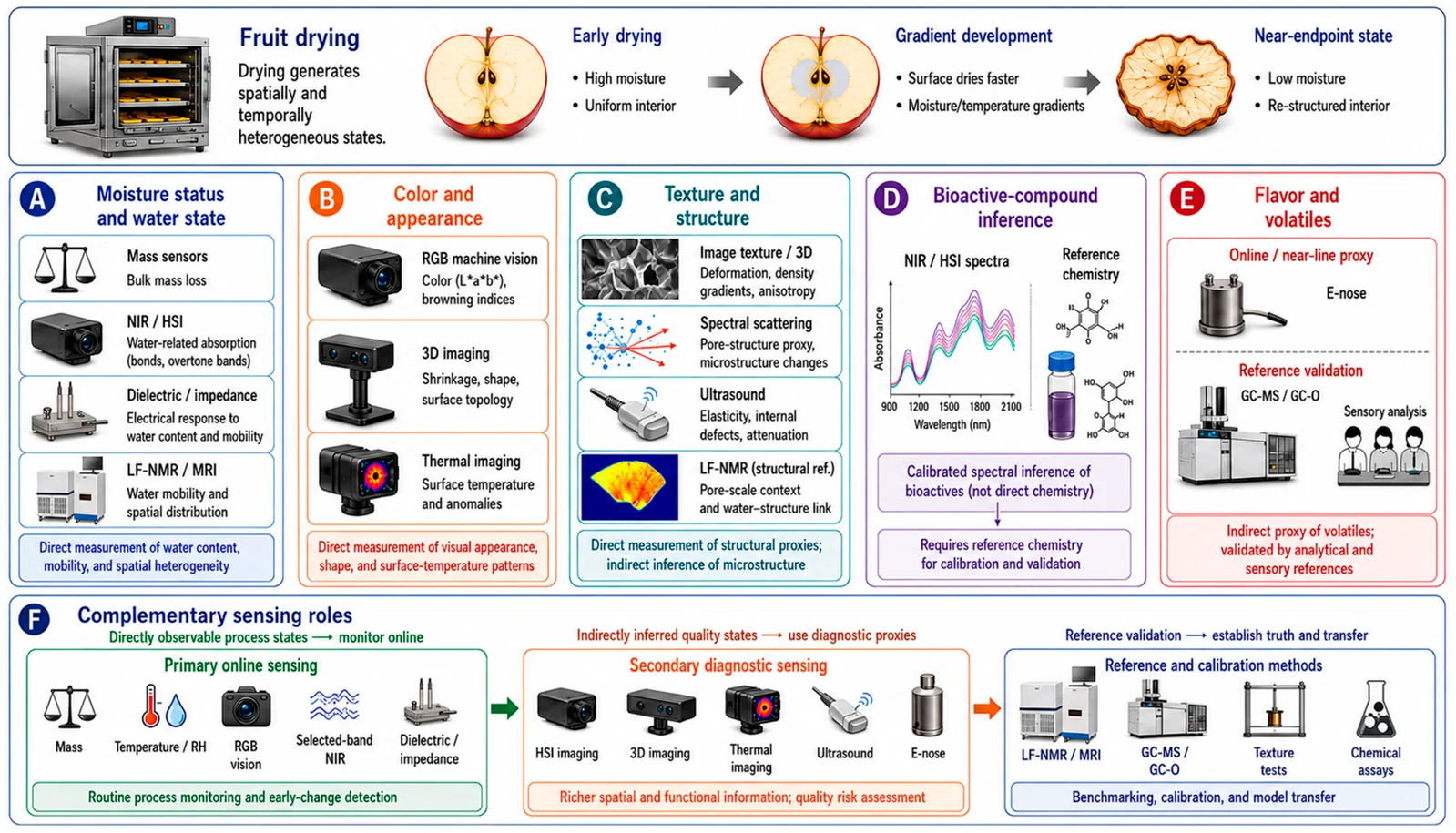
Schematic diagram of multimodal non-destructive sensing for monitoring moisture status, color and appearance, texture and structure, bioactive-compound-related responses, and volatile fingerprints during fruit drying. The magnetic resonance imaging (MRI) water-distribution images and scanning electron microscopy (SEM) micrographs were adapted from Ref. [73] with permission. Cropping, relabeling, and layout adjustments were performed for consistency.

Across these modalities, deployment simplicity trades off against access to internal, spatial, chemical, or sensory information (Figure 3). Table 2 summarizes quantitative performance reported in fruit-drying sensing studies.

Richer information generally carries greater requirements for acquisition, calibration, computation, or equipment (Table 3). Fast surface and process measurements are therefore suited to routine monitoring, whereas internal water, structure, composition, and aroma often require slower or more specialized methods. The useful balance depends on which part of the product state remains uncertain and whether the measurement supports routine monitoring, mechanistic diagnosis, or model maintenance.

## 5. Multimodal Sensing, Intelligent Prediction, and Model Reliability

### 5.1. Multimodal Fusion and Modality Selection

Multimodal fusion combines measurements at the data, feature, decision, or model level [121]. Raw-data fusion preserves detail but requires synchronized sampling volumes, scaling, and noise control. Feature-level fusion combines selected wavelengths, color, morphology, temperature, water-state, or volatile descriptors, while decision-level fusion reconciles predictions from separate models [130]. Joint architectures can learn interactions among modalities or targets. In fruit drying, feature- and decision-level approaches often retain a clearer relationship between each signal and the quality process it represents. Fusion is therefore most useful when the modalities observe complementary mechanisms at compatible times and locations [131].

Comparisons with single-modality models reveal whether fusion contributes new information. NIR–Raman fusion for salmon oxidation illustrates the value of complementary molecular signals in another food system [132]. During pulsed-vacuum drying, Fourier-transform near-infrared (FT-NIR) spectroscopy was more responsive to moisture, narirutin, and hesperidin, whereas Vis–NIR-HSI was more responsive to color; their fusion improved most predictions but not moisture, for which FT-NIR alone remained stronger [133]. Dual-modal sensing has likewise improved selected quality predictions when the signals described different aspects of the product [134]. These results show that fusion gains are target-specific. They may disappear when one input duplicates another, increases noise, or encodes batch- or instrument-specific variation. Apple drying provides a direct example of target-dependent fusion. In the band-pass-filter model, combining image features at 980 and 1450 nm produced a test-set *R*^2^ of 0.992 and RMSE of 2.21%, compared with *R*^2^ = 0.993 and RMSE = 2.45% at 1450 nm alone; the authors therefore found no significant improvement from combining the two channels for moisture prediction [105]. The result does not diminish the value of reduced-band imaging; it shows that an additional channel is useful only when it resolves uncertainty left by the first.

The combined action of ultrasound and cold plasma, for example, involves mechanical disruption, reactive chemistry, and structural sensitization that require different measurements [56]. Sustainability analyses similarly connect energy, nutrient retention, loss reduction, and scalability [3]. In each case, fusion becomes informative when the added signal explains a defined source of quality variation and the resulting model improves a relevant decision.

### 5.2. Mechanism-Oriented Feature Interpretation and Explainable AI

The variables selected by a model acquire meaning only when they can be related to the evolving product. Water-sensitive wavelengths reflect moisture and hydrogen bonding; color indices respond to pigments and browning; image texture and morphology reflect shrinkage and collapse; thermal features indicate heat load; and volatile fingerprints respond to aroma loss or oxidation. Because these changes co-vary with drying time, tray position, illumination, and sample geometry, a highly accurate model may still rely on a confounding feature [135,136]. Interpretation therefore begins with the physical origin of the signal rather than with the numerical importance assigned by the algorithm.

Shapley additive explanations (SHAP) and local interpretable model-agnostic explanations (LIME) attribute predictions to spectral or tabular variables, whereas gradient-weighted class activation mapping (Grad-CAM) and related methods locate influential image regions. In food models, these tools have revealed dependence on backgrounds, lighting, packaging, and device signatures rather than on the product itself [137]. XAI-guided wavelength selection has also reduced redundant inputs and visualized apple dry matter [117]. Statistical importance alone, however, does not establish chemical or physical meaning. A plausible feature must also remain stable across independent batches and instruments [138].

The value of explanation becomes most apparent when an accurate model uses the wrong information. In a spectroscopy study outside fruit drying, strong predictions persisted in chemically uninformative regions, and SHAP assigned importance to noise or instrument artifacts [139]. An analogous fruit-drying model might rely on detector-edge wavelengths, tray position, backgrounds, or illumination gradients instead of water-sensitive regions or tissue features. Masking tests, repeated acquisition, and cross-batch comparisons can expose such shortcuts. An implausible explanation is therefore evidence that the model requires revision, not merely an alternative visualization.

Interpretability is shaped throughout the modeling workflow. Calibration design, preprocessing, region-of-interest selection, feature extraction, and validation determine whether a model captures product chemistry or experimental artifacts [13,108,140]. Deep learning can reduce manual feature engineering, but it does not remove limitations arising from small datasets, uncertainty, or poor generalization [135,141]. Models become more useful when water-related wavelengths, browning indices, shrinkage features, volatile responses, and electrical variables can be connected to particular changes in the drying product. This connection supports both scientific interpretation and diagnosis when performance deteriorates.

### 5.3. Attribute-Specific Quality Prediction

#### 5.3.1. Moisture, Moisture Ratio, and Endpoint State

Moisture content, moisture ratio and drying endpoint are the most developed intelligent-prediction targets because they are directly related to process efficiency and product stability and can be referenced by standard gravimetric methods [23,128]. NIR, Vis-NIR, HSI, image analysis and mass measurements provide inputs for partial least squares (PLS), support vector regression (SVR), random forest (RF), artificial neural networks (ANNs), long short-term memory (LSTM) networks and related models [97,142]. However, endpoint prediction is not a simple regression task in which time or moisture dominates all other variables. A product may meet the target moisture value while showing excessive browning, poor rehydration, aroma loss or unnecessary energy consumption. AI-assisted drying reviews therefore frame endpoint prediction as a coupled process–quality task rather than a moisture-only regression problem [143]. Specific ANN-based and optimization studies on dragon fruit and kiwifruit further show that process variables and quality responses can be modeled together, but their decision value depends on whether quality consequences beyond moisture are evaluated [144,145].

Endpoint prediction changes as drying progresses. Early measurements describe rapid moisture removal and uneven heating; during the falling-rate period, internal resistance and structural change become more influential; near the endpoint, residual moisture must be considered together with color, texture, nutrients, aroma, and energy. HSI studies of microwave-vacuum vegetable drying and computer vision during tencha drying illustrate this time-dependent formulation outside direct fruit evidence [24,146]. In fruit, NIR has replaced destructive moisture assays during apple drying [96], while image and HSI measurements have added information about appearance and spatial heterogeneity [104]. Reliable endpoint models must retain this relationship when drying time, thickness, cultivar, or batch changes.

Moisture prediction is comparatively close to industrial use, yet it remains vulnerable to shortcuts [115]. A model trained under one drying schedule may learn elapsed time or temperature history instead of a transferable water response. Changes in cultivar, slice geometry, instrument, dryer, or the surface-core moisture gradient can then degrade performance [147]. NIR and machine learning show promise for online apple monitoring, and reduced-band multispectral systems offer a lower-complexity route to deployment [118]. Their practical value depends on uncertainty estimates and recognition of samples that lie outside the calibrated product and process range.

#### 5.3.2. Color, Browning, and Appearance

Color, browning and appearance prediction is relatively suitable for online use because the target signals are visible or surface-near [148,149]. Suitable outputs include L*, a*, b*, total color difference, browning index, shrinkage, defect class and appearance grade. RGB imaging provides a low-cost primary sensor for continuous monitoring, whereas HSI and 3D imaging separate spectral change, morphology and scattering when more diagnostic information is needed. Thermal descriptors can provide early warning of local heat stress before visible browning becomes pronounced. Prediction design needs to match the decision: regression is appropriate for color coordinates, whereas classification is generally more appropriate for categorical browning-severity or visible-defect grading.

Appearance can indicate drying state as well as surface quality, but the relationship is indirect. HSI signatures of dried jujube vary with cultivar and maturity [150], and research across machine vision and HSI shows that segmentation, illumination, and sample presentation alter color and shape features [18,151]. Browning models therefore need intermediate deterioration states and process variation, not only easily separated endpoints. Spectral or thermal information can add value when visual differences are subtle [12], although surface features still require caution when internal moisture or structure evolves differently.

A layered implementation strategy provides a practical balance between cost and information content. Continuous RGB imaging tracks color, area and shape; thermal imaging identifies heat-load anomalies; and selected spectral bands confirm pigment- or moisture-related changes [105,106]. Such systems are more deployable than full HSI but still require color calibration, illumination control and validation across cultivars and dryer designs [108]. Appearance prediction is mature for surface-quality monitoring, whereas reliable inference of internal quality requires complementary moisture, structural or chemical sensors.

#### 5.3.3. Texture, Rehydration, and Structural Quality

Texture, rehydration and structural quality are difficult prediction targets because they emerge from cell-wall integrity, pore architecture, residual water, glass transition and fracture behavior [22,152]. Two products with similar moisture contents may differ markedly in hardness, crispness or rehydration when one retains an open pore network and the other has collapsed [52]. The porous-scaffold model supports descriptors related to porosity, shrinkage, solid concentration and water state rather than time or moisture alone [49,86]. HSI and 3D imaging provide chemical and geometric information, but their relationship with mechanical texture must be calibrated against destructive or sensory reference measurements [101,111].

Texture models differ among fruits because the structures that govern fracture also differ. Image texture and microstructure distinguished mango cut types and osmotic or microwave treatments [50], while maturity-related cell-wall and water differences affected plum drying and quality [53]. Reviews outside fruit drying similarly show that firmness and internal structure depend on variety, sample orientation, and the reference test [14]. Geometry and raw-material descriptors therefore help explain why a model calibrated against one hardness or rehydration response may not retain the same meaning in another product.

For deployment, texture prediction is better treated as a secondary or confirmatory layer than as a fully direct online measurement [110]. Conventional texture analysis provides reference values but is destructive and spatially limited [111]. Ultrasound-based analysis provides internal structural information because acoustic velocity, attenuation and impedance respond to density gradients and interfaces [112], while NIR and HSI capture water- and composition-related correlates of texture [108]. Rehydration ratio adds information about pore-network and cell-wall damage history. A robust framework uses mechanical or sensory endpoints as labels while combining water mobility, surface shrinkage and internal-porosity features.

#### 5.3.4. Bioactive Retention and Flavor Quality

Bioactive-quality prediction is more difficult than moisture or appearance prediction because reference assays are costly, destructive, and chemically heterogeneous [135]. NIR or HSI models may estimate phenolics, flavonoids, vitamin C, or antioxidant activity, but they must separate true chemical information from correlations with water, color, browning, and scattering. Metabolomics of dried noni showed that different drying methods produced distinct active-metabolite profiles, suggesting that profile-level or multi-output prediction is more informative than a single total-content value [58]. Orange-peel and kiwifruit studies likewise show that phenolics, flavonoids, vitamin C, antioxidant capacity and rehydration do not change in parallel [41,51]. Bioactive models require rigorous reference chemistry, variation across relevant batches and operating conditions, and independent validation; at present, they are better suited to screening and quality-risk warning than to universal quantification [61,115].

Flavor prediction is even more sensitive to interpretation because volatile abundance, aroma activity and sensory perception are not equivalent [153,154]. Electronic-nose fingerprints are suitable for rapid classification or trend monitoring, while GC-MS, GC-olfactometry and sensory analysis assign compound-level and perceptual meaning. For consumer-oriented prediction, an electronic-nose warning or a total volatile peak-area model should be treated as a proxy unless it is calibrated against odor-activity information, GC-olfactometry, or sensory labels. Studies of sapodilla, raspberry, and jujube show that drying selectively removes, generates, or transforms aroma-active compounds and that volatile profiles continue to evolve during storage [64,65,66]. Cold-plasma research further demonstrates that operating parameters and matrix composition shift volatile classes in desirable or oxidation-related directions [26]. More informative models combine gas-sensor fingerprints with chromatographic markers, process variables, and sensory labels rather than training solely on total volatile abundance [121,155].

### 5.4. Multi-Quality Prediction and Decision Support

Fruit drying rarely has a single optimum because storage stability, color, texture, nutrients, characteristic aroma, energy demand, throughput, and cost respond differently to the same operating condition [156]. Moisture or water activity, under-drying risk, and unacceptable thermal or microbial damage define the feasible region rather than ordinary preference scores [45,127]; hazard-related outcomes likewise require their own targets and reference methods [157]. Within that region, neural-network optimization of dragon fruit and kiwifruit demonstrates how kinetics and quality responses can be balanced [144,145], but the preferred solution changes with the product and process [87,90]. Crisp snacks place greater value on color and fracture behavior, functional ingredients on bioactive retention, and industrial lines on throughput and energy [1,158]. Comparisons of electrohydrodynamic and other physical-field methods similarly show that process and product indicators must be interpreted together [71,92,159].

The difficulty in multi-quality prediction lies in representing conflict among outcomes rather than in increasing their number [39]. Drying time, energy, color, texture, rehydration, aroma, and nutrient retention rarely change in the same direction, so an aggregate score can conceal the loss of an important attribute [17]. Multi-output fruit assessment offers methods for separating constraints, preferences, and diagnostic variables, although the roles must be defined for the intended dried product [160]. Optimization can then identify feasible operating regions, but the selected point remains dependent on the assigned quality and resource priorities. Useful models therefore report uncertainty and the sensitivity of the decision to weighting assumptions.

**Figure 4:**
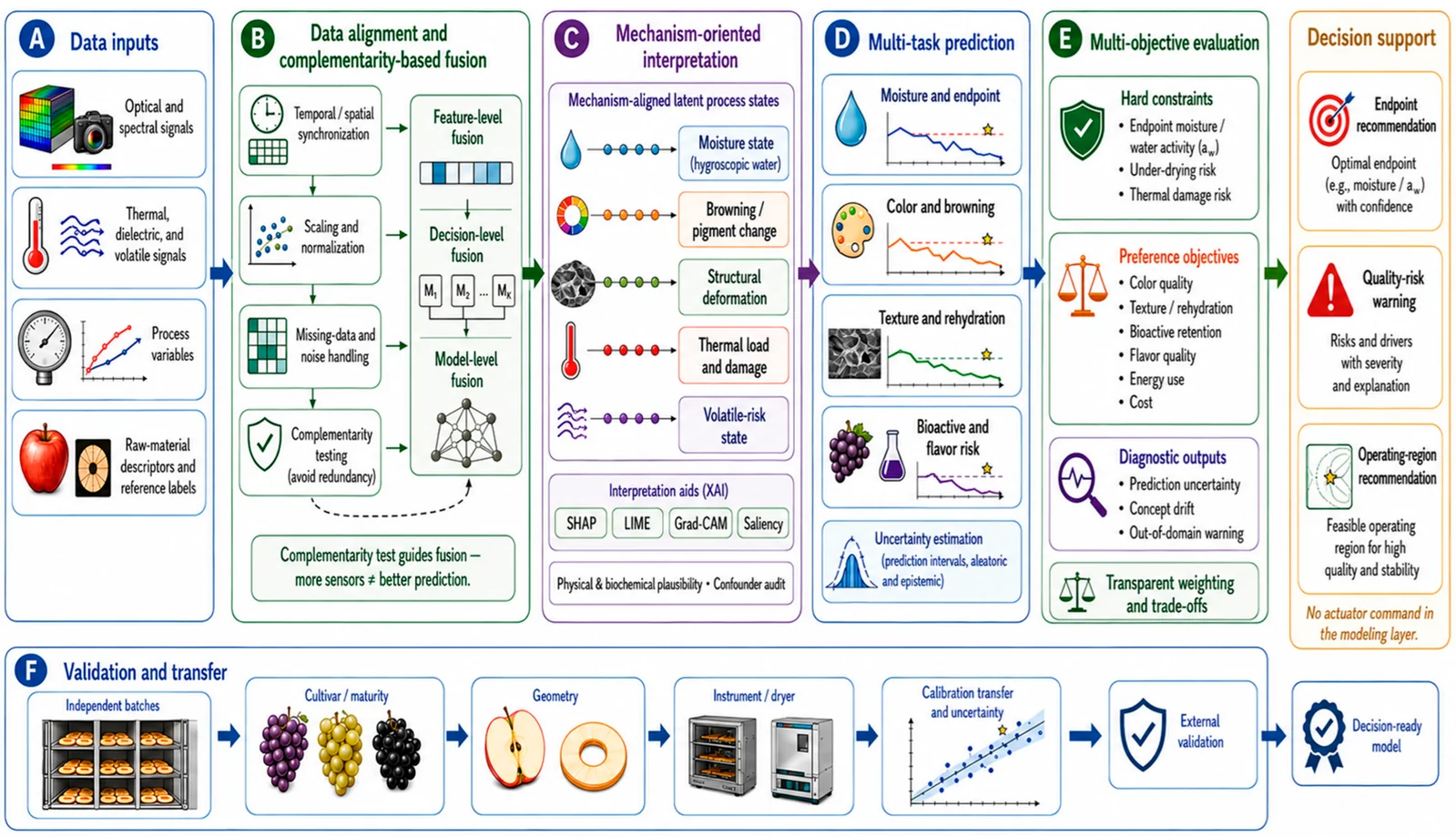
Schematic diagram of multimodal data fusion and intelligent prediction of fruit-drying quality, integrating data alignment, complementarity-based fusion, mechanism-oriented interpretation, multi-task prediction, multi-objective evaluation, decision support, and model validation and transfer. The microstructural image panels were reproduced or adapted from Ref. [73] with permission. Cropping, relabeling, and layout adjustments were performed for consistency.

Prediction tasks differ in the information and reference measurements needed to support a decision (Table 4). Research in precision agriculture and fruit assessment shows that fused sensors can estimate maturity, sugars, acidity, and firmness, but field and temporal variability affect model validity [161]. Spectral reviews similarly identify feature selection and validation as determinants of performance beyond the calibration set [162]. During drying, a moisture model may identify a stable endpoint while overlooking aroma loss [153,154]. Decision support must therefore preserve the individual meanings and uncertainties of moisture stability, appearance, texture, bioactive retention, flavor risk, energy, and process feasibility. Figure 4 brings these relationships into a common prediction and decision framework.

### 5.5. Model Reliability, Validation, and Transferability

Model transfer is difficult because both the fruit and the sensing environment vary [147,166]. Cultivar, maturity, slice geometry, pretreatment, soluble solids, firmness, surface wax, and tissue structure alter the relationship between signal and quality [125,150,158]. Instrument replacement, illumination, temperature, humidity, and sample presentation introduce additional shifts [97]. Drying mode further changes the relationship between surface and internal state, so a hot-air calibration may not retain its form in microwave, vacuum, or hybrid drying. Reviews of fruit spectroscopy and related root-and-tuber sensing consistently show that robustness across seasons, cultivars, instruments, and matrices must be tested rather than inferred from a random split of one dataset [84,136].

Transfer improves when these sources of variation are represented rather than discarded. Stable features, standardization samples, calibration transfer, domain adaptation, and transfer learning can reduce batch and instrument effects [78,162,167]. Geometry, maturity, dry matter, process history, and sensor configuration also belong in the metadata or model when they explain signal variation. Reference texture methods require similar standardization across sites and instruments [111]. Similar domain shifts are documented in precision agriculture, tuber grading, and general food sensing. For fruit drying, external tests across cultivars, seasons, instruments, or dryers provide a more realistic estimate of deployment performance than records drawn from the calibration experiment.

Drying datasets also contain repeated observations of the same fruit, slice, batch, or run. Those records share product identity and process history, so distributing them across training and test sets leaks information. Leakage may also enter when preprocessing, feature selection, response-informed splitting, or hyperparameter tuning is performed before the test fold is isolated [168]. Resampling should therefore keep independent fruits or drying batches together, with all modeling choices made within the training data. A separate cultivar, season, instrument, or dryer can then provide the final transfer test.

## 6. Process Analytical Technology and Deployment for Sensor-Based Fruit Drying

### 6.1. From Offline Reference Analysis to In-Line and Online PAT

Offline assays establish the reference meaning of moisture, texture, composition, and aroma, but their sampling interval is too slow to follow the changing state of a drying batch [131]. Machine vision, NIR, thermal, electrical, mass, and environmental sensors can close this temporal gap [93]. In jujube drying, machine vision and automatic weighing followed color and moisture in real time, with a mean relative error of 0.18% for moisture content; the a* coordinate served as a calibrated proxy for vitamin C and reducing sugar [129]. The system illustrates a practical intermediate stage: the product state becomes visible during operation, yet the estimate does not itself adjust the dryer. Progress from monitoring to control therefore depends on synchronized reference data, stable acquisition under heat and humidity, and an explicit link between each prediction and a process action [169].

Conditions inside a dryer determine whether a laboratory sensor remains reliable [18,85]. Condensation and deposits obscure optical windows; movement, vibration, and illumination alter image signals; heat and humidity accelerate drift; and computation must fit the interval available for control. Models also need to recognize observations outside their calibration domain and communicate uncertainty [161]. Fruit and spectral studies show why calibration transfer and stable measurement geometry matter across instruments and products [14,78]. An industrial system can therefore begin with a small set of maintained signals and expand when another modality resolves a specific operational uncertainty [166].

PAT links process conditions and product attributes through maintained measurements, time-resolved interpretation, and a rule for intervention. Chamber temperature, relative humidity, airflow, pressure, mass, and energy describe the dryer, while RGB, thermal, NIR, electrical, or volatile measurements describe the product. Soft sensors combine these streams to estimate variables that would otherwise require destructive analysis [170]. Most food-drying systems, however, still stop at monitoring or prediction. The transition to PAT occurs when an estimate changes an operating decision, initially through an operator recommendation and, after validation, through a bounded automatic action.

### 6.2. From Monitoring to Multi-Quality Decision Support

Control decisions differ from quality predictions because some outputs define limits while others express preferences [171]. Endpoint moisture or water activity, excessive product temperature, severe browning, case hardening, under-drying, and storage-related microbial risk constrain acceptable operation [1,127]. Drying time, energy, color, crispness, rehydration, nutrient retention, and characteristic aroma can then be optimized within that region [144,172]. Rapid temperature rise, abnormal shrinkage, volatile drift, or divergence between surface and internal moisture provide warnings that the process is approaching an undesirable state. This separation prevents an improvement in one score from concealing loss of stability or product quality.

These layers form a progression from measurement to intervention: sensing updates the product state, prediction estimates the endpoint and emerging quality risks, and the controller adjusts the process within defined operating limits [129,163,164]. When moisture remains high and thermal risk is low, drying can continue. Rising surface temperature or browning risk may instead favor a lower heat load or a change in stage, while an approaching endpoint accompanied by texture or aroma deterioration may favor termination. Available studies support individual parts of this sequence, but not yet a transferable autonomous loop for fruit drying. Operational use therefore requires each prediction to be linked to a defined actuator range, response time, and fail-safe action.

### 6.3. Bounded Model-Assisted Control and Operational Safety

Model-assisted control adjusts drying conditions according to estimated product state rather than a fixed time-temperature schedule [163,173]. Manipulated variables include air temperature, relative humidity, and air velocity in hot-air drying; microwave or radio-frequency power, pressure, and pulse ratio in microwave or vacuum drying; radiation intensity and source-sample distance in infrared drying; and stage-switching points in hybrid drying. State variables include moisture, water activity risk, surface temperature, browning risk, shrinkage and structural quality. AI-assisted drying and optimization studies show that neural networks and related models capture nonlinear relationships between operating conditions and product responses [143,145,174]. Advanced transport models of anomalous diffusion [165], together with digital-twin and closed-loop frameworks [175], provide methodological evidence for coupling sensors, physicochemical models and control decisions. Transfer to fruit drying nevertheless requires validation under fruit-specific shrinkage, heterogeneity, heat sensitivity and batch variability. Control performance should be evaluated under disturbances and batch variation, not inferred only from offline prediction accuracy.

Control performance depends on synchronized sensing, bounded actuation, and verification of the resulting product. Signal-quality and domain checks precede state estimation; the model then predicts the current condition and near-future risk, and the controller selects an adjustment within safe limits. Latency, uncertainty, and fail-safe behavior can be as important as average prediction accuracy. Physics-based or structural constraints may reduce implausible estimates and help distinguish product change from sensor drift. Near-term systems are therefore likely to support operators before progressively assuming autonomous control [164].

### 6.4. In-Line Deployment and Readiness of Embedded Multisensor Systems

Embedded systems combine measurements of the drying environment with measurements of the product [129]. Temperature, humidity, airflow, mass, pressure, and energy describe operating conditions, while RGB or thermal images, NIR or multispectral signals, impedance, and volatile patterns describe product state [95]. Together, these signals need to resolve moisture progression, thermal risk, and the quality attribute that limits the intended product [102,176]. Digital-twin or soft-sensor models can then update the estimated state. The sensor set follows from the endpoint or intervention to be supported, rather than from the number of instruments available.

The embedded pipeline synchronizes measurements, checks signal quality, extracts or fuses features, estimates state, and forecasts endpoints or risks [175]. Edge processing, model updating, fault detection, and readable interfaces reduce the delay between measurement and action [131]. Because drying proceeds more slowly than most sensors sample, windowed aggregation, selected-band acquisition, adaptive sampling, and event-triggered updates can reduce data load without losing meaningful changes. Rapid events such as local overheating still require a sensing-to-decision delay shorter than the control interval. Manual override, alarm limits, post-drying verification, and traceable records remain part of the control system [16].

**Figure 5:**
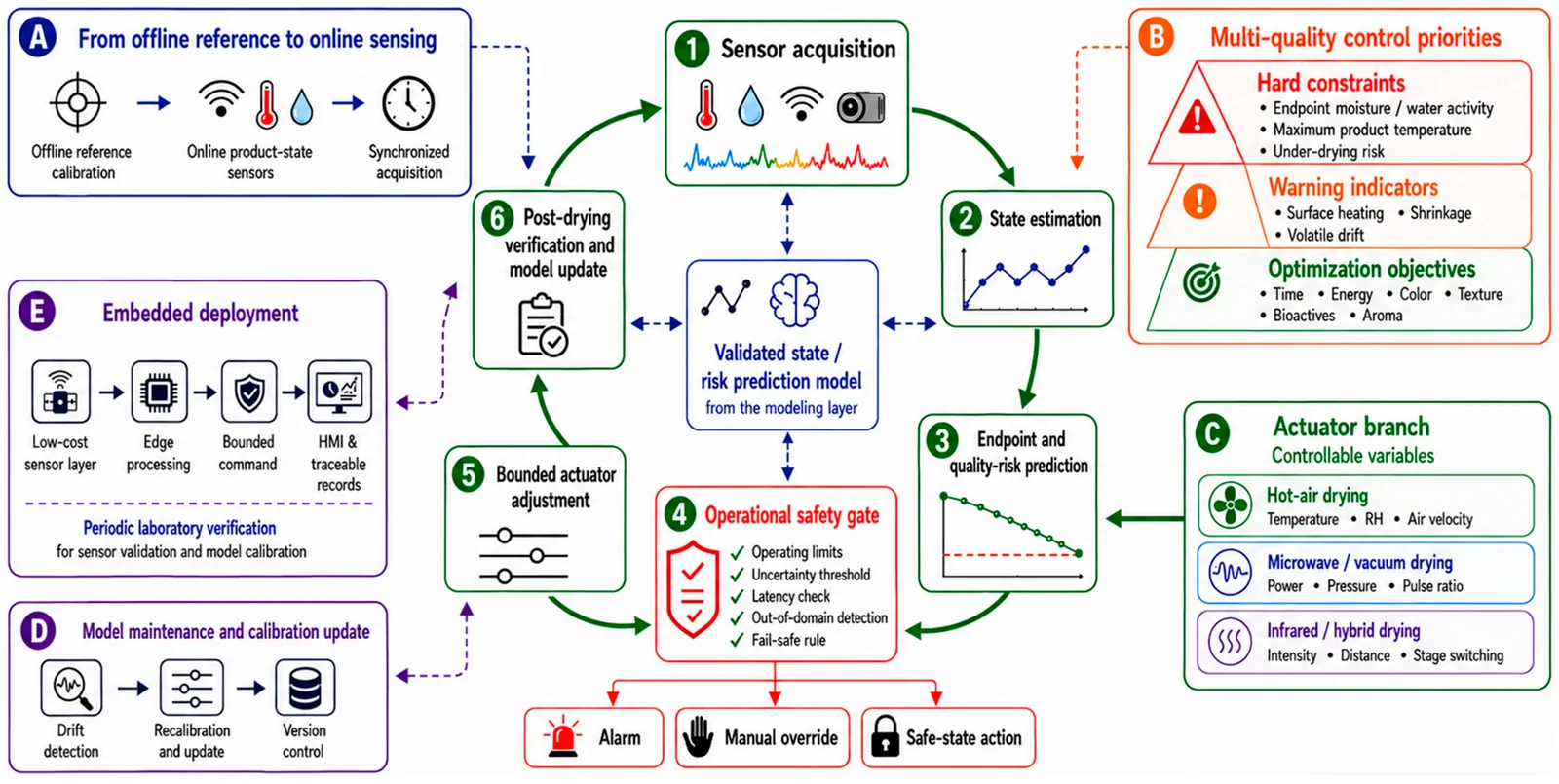
Schematic diagram of sensor-guided adaptive control for fruit drying, integrating online sensing, state estimation, endpoint and quality-risk prediction, safety-constrained actuator adjustment, post-drying verification, embedded deployment, and model maintenance.

Cost, cleaning, fouling, drift, calibration maintenance, data infrastructure, and operator training constrain industrial adoption [85]. These burdens can offset favorable laboratory performance, especially in smaller facilities [8]. A modular system can begin with RGB, temperature, humidity, and mass measurements and add selected NIR or multispectral bands when composition-related information changes a decision [98,118]. More expensive modalities can support calibration, troubleshooting, or high-value products, while electrical sensing can add bulk-moisture information when contact, geometry, and temperature are controlled. The resulting adaptive-control concept is shown in Figure 5.

Batch and continuous dryers impose different measurement geometries. Fixed cameras or optical probes can repeatedly observe trays sampled across defined dryer positions in a batch cabinet together with chamber and mass measurements. In a conveyor or tunnel dryer, the product moves through residence-time zones, so sensing must be synchronized with belt speed or product tracking and provide adequate cross-belt coverage. Optical access, cleaning, drift checks, and outlet reference measurements become part of the measurement chain. Continuous infrared-assisted hot-air drying of garlic shows how product-flow data can be coupled with quality prediction [174], but the same arrangement has not yet been validated across fruit products. Pilot studies must determine whether changing load, geometry, cultivar, or belt distribution alters the residence-time map and the resulting control decision.

Sensor roles also change along the production sequence. Before drying, rapid measurements describe raw-material variability and support grading, loading, and initial process selection. During drying, repeated measurements reveal the evolving product state and support endpoint or risk decisions. After drying, outlet and reference measurements confirm stability, detect residual non-uniformity, and provide data for model maintenance. Table 5 organizes these functions across offline, at-line, online, and in-line deployment.

Real-time mass, moisture-related signals, color, and selected quality attributes can already be observed during fruit drying, as illustrated by the jujube system in Section 6.1. Near-term systems can therefore combine maintained temperature, humidity, mass, RGB, and selected-band NIR measurements with operator-facing endpoint and risk estimates. Electronic-nose, electrical, hyperspectral, or fused loops become worthwhile only when their added information offsets latency, cleaning, and maintenance. LF-NMR, MRI, GC–MS/GC–O, and destructive texture or chemical tests remain valuable for calibration and periodic verification. Closed-loop frameworks define the longer-term architecture, but transfer across fruits and dryers will depend on actuator experiments that show how a predicted state changes the product [164].

## 7. Conclusions and Future Outlook

Fruit-drying quality develops along a trajectory rather than at a single endpoint. Moisture migration changes water state and structure, while thermal and oxidative histories reshape pigments, nutrients, and aroma. The resulting balance varies with species, cultivar, maturity, tissue geometry, drying method, and pretreatment. A short drying time or acceptable final moisture content therefore does not alone establish product quality.

Non-destructive sensing makes parts of this trajectory observable. Vision and thermal imaging describe surface appearance and heat distribution; NIR and spectral imaging reveal moisture-related and selected compositional changes; LF-NMR/MRI resolves water mobility; electronic noses follow volatile fingerprints; and electrical measurements add information about bulk state. Prediction models can turn these signals into endpoint estimates and quality-risk warnings, but their value depends on whether the measured signal retains the same meaning across products, batches, instruments, and dryers.

The next stage of intelligent fruit drying is therefore less about adding sensors than about establishing reliable relationships among product state, prediction, and intervention. Shared datasets should retain fruit characteristics, geometry, process history, synchronized signals, and reference measurements so that models can be tested across cultivars, seasons, instruments, and dryers. Transferable calibration, uncertainty estimates, interpretable features, and maintained sensing systems will determine whether laboratory predictions remain useful in production. Pilot-scale control studies must then show that an intervention preserves the intended balance of water removal, structure, nutrients, aroma, energy, and throughput. In continuous dryers, this requires measurements that remain associated with the moving product and its residence-time zone, together with outlet verification. These developments would move the field from isolated demonstrations of prediction toward traceable and product-specific process control.

## Figures and Tables

**Table 1:** Scope and positioning of the present review.

Literature Stream	Coverage in Previous Reviews	Remaining Gap for Process–Quality Integration	Contribution of the Present Review
Drying technologies and pretreatments	Emerging thermal and nonthermal processing, physical-field drying, freeze drying, heat-pump drying, and pretreatment-assisted dehydration have been summarized mainly from efficiency, energy, and endpoint-quality perspectives [2,3,6].	The literature is often organized by technology type, which can weaken the explanation of how water transport, tissue deformation and chemical reactions jointly form quality.	Reorganizes the evidence around moisture migration, structural remodeling, reaction pathways and quality coupling during fruit drying.
Quality evolution and quality-change modeling	Bioactive retention, color, texture, microbial stability and dried-product texture formation have been reviewed as major quality dimensions of fruits and vegetables [22].	Many discussions remain attribute-specific or endpoint-oriented, while interactions among water state, matrix structure, nutrient release/degradation and flavor remodeling are less integrated.	Builds a process-oriented framework linking water state, microstructure, color, texture, bioactives and volatile profiles.
Non-destructive sensing for drying and fruit quality	Fruit-drying studies have applied machine vision, NIR/Vis-NIR, HSI, and LF-NMR/MRI to moisture, color, morphology, and selected chemical attributes [23,24,25].	Single-modality studies commonly target one attribute, and limitations such as scattering, sensor drift, calibration transfer and online deployability are not always evaluated together.	Maps each sensing modality to the physical or chemical information it can reasonably represent, while emphasizing complementarity and deployment constraints.
AI, data fusion and sensor-based drying control	AI-assisted drying, multi-objective optimization and edge-enabled non-destructive sensing have been reviewed as routes for endpoint detection, quality-risk warning and adaptive process decisions [16,17,18].	Prediction accuracy is often discussed separately from external validation, model transfer, uncertainty, sensor maintenance and control feasibility.	Relates complementary sensing signals and, where justified, fused features to interpretable prediction, multi-objective evaluation, endpoint decisions, and quality-risk warnings for future drying-control systems.
Mechanism-oriented cold-plasma pretreatment literature	Cold-plasma drying reviews emphasize surface etching, microstructural modification, moisture diffusion, and energy reduction [7].	These reviews provide strong mechanistic insight into pretreatment-assisted drying, but are less focused on how the resulting quality changes can be monitored and predicted.	Uses pretreatment mechanisms as mechanistic examples linking structural remodeling, water migration, oxidative reactions, and quality-risk prediction.
Flavor- and sustainability-oriented drying literature	Flavor reviews connect processing conditions with volatile rearrangement and aroma shifts, whereas sustainability reviews connect drying with food security, waste valorization, clean energy, and circular systems [8,26].	Flavor is often treated separately from drying kinetics, and sustainability is frequently discussed separately from product-quality sensing and online control.	Integrates aroma transformation and sustainability considerations into a broader process–quality framework for sensor-based fruit-drying control.

**Table 2:** Quantitative performance reported in fruit-drying sensing studies.

Fruit and Drying Context	Sensing and Acquisition	Sampling and Validation	Target and Model	Best Reported Performance	Ref.
Kiwifruit slices; pretreatment-assisted drying	RGB machine vision; Nikon camera, 4288 × 2848 px; 5500 K illumination; images every 30 min	2700 image-time records; 2459 retained after exclusions; five-fold cross-validation	Moisture ratio; random forest	*R*^2^ = 0.9923; RMSE = 0.0312; MAE = 0.0211	[104]
Apple slices; ultrasonic drying	Portable NIR, 900–1700 nm; 228 wavelengths; 32 scans per sample	142 samples; leave-one-out cross-validation and an independent external set	Moisture content; SPA–GPR	*R*^2^_p_ = 0.991; RMSEP = 2.841%	[23]
Jujube slices; hot-air drying at 55, 60, and 65 °C	HSI, 400–1000 nm; 228 bands; 4.69 nm resolution; 23.2 s per cube	270 samples; Kennard–Stone split, 190 calibration and 80 prediction; ten-fold cross-validation for tuning	Moisture, hardness, soluble solids content (SSC), and color; SVR	Moisture: *R*^2^_p_ = 0.935, RMSEP = 1.896; hardness: 0.955, 1.476; SSC: 0.941, 1.732	[25]
Apple slices; freeze drying at five sampling times	Portable NIR transflectance, 900–1700 nm; 701 points; 90 repeated scans	300 spectra at each time point; sample-set partitioning based on joint *x*–*y* distances (SPXY) for calibration/prediction splitting and cross-validation; spectra were repeated measurements rather than 1500 independent fruits	Wet-basis moisture content; SG–SNV–SPA–RFR	*R*^2^_p_ = 0.9876; RMSEP = 0.0293	[96]
Apple slices; convective drying	NIR monochrome imaging at 980 and 1450 nm using LED and band-pass-filter configurations	126 measurements per attribute and imaging series; 75/25 development split plus apples from an external source	Moisture content; GPR	External light-emitting diode (LED) models: 1450 nm *R*^2^ = 0.991, RMSE = 3.49%; 980 nm *R*^2^ = 0.987, RMSE = 3.11%	[105]
Apple slices; convective drying	Laser-light backscattering and biospeckle imaging at 635, 980, and 1450 nm	252 slices; 126 moisture records; 75/25 split, ten-fold CV, and 36 external measurements	Moisture and selected quality attributes; GPR	External laser-light backscattering imaging (LLBI) at 1450 nm: moisture *R*^2^ = 0.95, RMSE = 6%; vitamin C *R*^2^ = 0.91, RMSE = 0.69 g 100 g^−1^ fresh weight (FW)	[106]
Mango slices of three maturity stages; drying at three temperatures	Laser backscattering imaging at 450, 520, and 635 nm	27 mangoes × 3 slices = 81 slices; MLR fitted across maturity and temperature conditions	Moisture content; MLR	Best at 635 nm: *R*^2^ = 0.9247; RMSE = 0.0771	[128]
Jujube slices; real-time hot-air drying validation at 65 °C and 4 m s^−1^	Machine vision plus automatic weighing	Continuous validation along the drying trajectory	Moisture, L*, a*, b*, vitamin C, and reducing sugar proxies	Moisture mean relative error = 0.18% (maximum 0.71%); mean L*, a*, b* errors = 0.93, 0.52, 0.73	[129]
Jujube; drying under multiple operating conditions	Vis–NIR spectroscopy, 325–1075 nm; 3.5 nm optical bandwidth; three spectra at different rotations	Ten-fold cross-validation	Moisture ratio; multilayer perceptron	Reported correlation *R* = 0.9968 (not *R*^2^); RMSE = 0.0074; MAE = 0.0046	[97]

Note: R^2^, coefficient of determination; R^2^_p_, coefficient of determination for the prediction set; RMSE, root mean square error; RMSEP, root mean square error of prediction; MAE, mean absolute error; SPA, successive projections algorithm; GPR, Gaussian process regression; SG, Savitzky–Golay; SNV, standard normal variate; RFR, random forest regression; MLR, multiple linear regression; SVR, support vector regression.

**Table 3:** Comparative characteristics and deployment roles of principal sensing modalities for fruit-drying quality assessment.

Sensing Modality	Measured Information and Effective Sampling Region	Acquisition Mode and Throughput	Role in Fruit-Drying Assessment	Deployment Requirements and Current Practical Use
Machine vision	Surface color, area, shape, shrinkage, wrinkling, and visible defects; full-field surface measurement.	RGB images or video; rapid area coverage, with throughput governed by illumination, exposure, and product presentation.	Continuous surface surveillance, browning warning, shrinkage tracking, and assistance with moisture or endpoint prediction [104].	Relatively low hardware and integration burden; suitable for online/in-line use when lighting, color calibration, and viewing geometry are controlled.
Near-infrared spectroscopy (NIR)	Water- and composition-related absorption from a spot or averaged near-surface sampling volume; penetration depends on wavelength and matrix.	Point or line acquisition; rapid spectra are compatible with repeated at-line or online measurements.	Moisture estimation, endpoint support, and calibrated screening of selected chemical attributes [23].	Moderate integration burden; portable and selected-band instruments are closer to deployment than full laboratory spectrometers, but calibration must be maintained across batches and instruments.
Hyperspectral imaging (HSI)	Spatial–spectral information on surface and near-surface heterogeneity, including moisture-, color-, and composition-related responses.	Area or line-scan image cubes; slower acquisition and heavier data processing than RGB or point NIR.	Mapping non-uniform dehydration and predicting multiple quality attributes in controlled fruit-drying studies [25].	Useful for laboratory and pilot-scale diagnosis; in-line use requires controlled illumination, motion synchronization, wavelength reduction, and real-time processing.
Thermal imaging	Surface temperature and its spatial distribution; no direct measurement of internal moisture or chemistry.	Rapid, non-contact full-field imaging; compatible with continuous monitoring.	Detection of uneven heating and local thermal risk; complements RGB or spectral sensing for browning warning [93].	Suitable for online/in-line surveillance when emissivity, reflections, viewing angle, and optical-window condition are controlled; fruit-drying studies report fewer directly comparable prediction metrics than for NIR or HSI.
Low-field nuclear magnetic resonance (LF-NMR)	Bulk water mobility and proton populations; ensemble information from the measured sample volume.	Intermittent benchtop acquisition; slower and less accessible than optical monitoring.	Mechanistic interpretation of water-state redistribution and its relation to shrinkage or texture [37].	Best suited to offline or at-line reference measurements; magnet size, sample handling, and environmental requirements limit dryer integration.
Magnetic resonance imaging (MRI)	Spatially resolved internal water distribution and structural heterogeneity throughout the sample volume.	Volumetric imaging with relatively long acquisition and high instrumentation demand.	Visualization of internal moisture gradients and validation of surface or proxy measurements [73].	Primarily a laboratory reference method; high cost, space, and integration requirements make routine in-line deployment impractical.
Electronic nose	Headspace volatile fingerprint from a gas-sensor array; response reflects the combined effects of volatiles, humidity, and background gases.	Repeated headspace measurements; response and recovery times depend on sampling flow and sensor conditioning.	Warning of odor-space shifts, oxidation, or off-flavor risk rather than compound-specific aroma quantification [120].	Potentially lower-cost than chromatographic analysis, but online use requires controlled sampling, humidity compensation, drift management, and periodic chemical or sensory validation.
Dielectric/impedance sensing	Electrical response associated with water content, ionic mobility, temperature, contact, and electrode geometry; local or bulk sensitivity depends on probe design.	Rapid point or embedded measurement; contact or near-contact acquisition.	Complementary moisture-state indication and endpoint support when geometry and temperature effects are controlled [95].	Potentially compact and inexpensive for embedded use, but fruit- and fixture-specific calibration and hygienic probe integration remain necessary.

**Table 4:** Prediction tasks and decision relevance for sensor-guided fruit drying.

Prediction or Decision Task	Typical Inputs and Modeling Approaches	Evidence Discussed	Decision Relevance and Limitation
Moisture ratio, moisture content and endpoint prediction	NIR/Vis-NIR/HSI spectra, image features, mass loss and process variables; PLS, SVR, RF, ANN, convolutional neural networks (CNNs), long short-term memory (LSTM) networks, and empirical or hybrid models.	Vis-NIR and machine-learning models have been used for moisture-ratio prediction of jujube [97], NIR was used for apple drying-moisture evaluation [23], and machine-learning interfaces have been developed for pomelo-peel drying [163].	Endpoint prediction should be linked to quality stability and under-drying risk rather than final moisture alone.
Color, browning and appearance grading	RGB features, HSI spectra, thermal descriptors and drying history; partial least squares discriminant analysis (PLS-DA), support vector machines (SVMs), RF, CNNs and other neural networks.	Image features predicted kiwifruit moisture and supported appearance-quality evaluation [104], HSI predicted hot-air-dried jujube quality parameters [25], and neural-network models predicted color changes in solar-dried fruits [148].	Models need to distinguish true browning or pigment degradation from correlations with drying stage, illumination or shrinkage.
Texture, shrinkage, porosity and rehydration prediction	Image texture, 3D geometry, shrinkage descriptors, LF-NMR and ultrasound features, and process variables; regression, ensemble learning and multi-task models.	Texture reviews emphasize integration of pore/cell morphology, solids and water state [22]; LF-NMR studies connect water state with shrinkage [37], and ultrasound provides an additional internal-structure proxy [110].	Texture models require structural evidence and mechanical or sensory reference tests; moisture alone is an incomplete predictor.
Bioactive-quality prediction	NIR/HSI spectra, process history and destructive chemical-reference data; PLS, RF, extreme gradient boosting (XGBoost), CNNs and multi-output regression.	Drying studies reveal method-specific active-metabolite profiles [58], while spectral-regression reviews describe the potential of NIR/HSI models for chemically related quality attributes [115].	Predictions are often indirect and require rigorous reference chemistry, independent validation and checks against moisture-, color- or scattering-driven confounding.
Flavor and volatile-risk prediction	Electronic-nose fingerprints, GC–MS markers, process and storage history, and sensory labels; classification, regression and multimodal fusion.	Flavor-prediction reviews emphasize integrated chemical and sensory data [153]; jujube and goji-berry studies show process- and storage-dependent volatile changes [66,154].	Gas-sensor or chromatographic outputs need aroma-activity, GC–O or sensory validation before they are interpreted as consumer-relevant flavor quality.
Data fusion and feature interpretation	Feature-level or decision-level fusion of spectral, image, volatile and process data; competitive adaptive reweighted sampling (CARS), genetic algorithms (GAs), SHAP, and XAI, attention models and graph-based fusion.	Yang et al. integrated FT-NIR and Vis-NIR-HSI for rapid prediction of critical quality attributes during pulsed-vacuum drying [133]. Arrighi et al. reviewed the diagnostic use and limitations of XAI in food models [137], and Zhang and Yang emphasized calibration transfer and robustness in fruit spectral analysis [136].	Fusion should be problem-driven; adding modalities without complementary information or feature interpretation can increase cost and reduce robustness.
Multi-objective optimization and control recommendations	Predicted quality indices, energy use, drying time, quality-stability constraints and control variables; artificial neural network–genetic algorithm (ANN–GA) optimization, Bayesian optimization, digital twins, physics-informed neural networks (PINNs), and model-predictive control.	AI-based multi-objective optimization, intelligent monitoring and closed-loop drying-control reviews call for integration of sensing, prediction and actuation [16,102,164]. Physics-informed or hybrid models may improve physical consistency in drying prediction [165].	Optimization can generate control recommendations, but closed-loop actuator adjustment in fruit drying still requires independent validation, uncertainty handling, safe operating rules and latency-aware implementation.

**Table 5:** Stage- and deployment-specific organization of single- and multimodal sensing in fruit-drying workflows.

Drying Stage	Measurement Mode	Sensor Configuration and Location	Information Generated	Process or Quality Decision
Before drying	Offline/at-line	Laboratory or near-line RGB/3D imaging, NIR/HSI, and reference measurements on raw-material samples drawn across the incoming lot.	Initial color, geometry, maturity, composition, and within-lot variability.	Raw-material grading, exclusion of atypical material, calibration design, and selection of initial drying conditions [166].
Before drying	Online/in-line	Conveyor-mounted RGB or selected-band NIR/multispectral sensing upstream of dryer loading.	Size, shape, surface condition, and rapid compositional proxies for individual items or lots.	Sorting, loading standardization, and assignment of product-specific initial set points [118].
During drying	Offline/at-line	Periodically removed samples assessed by gravimetry, texture or chemical tests, HSI, LF-NMR, or MRI near the dryer.	Reference moisture, water mobility, internal gradients, structural change, and chemical quality.	Calibration and validation of online proxies, diagnosis of surface-core divergence, and model maintenance [37].
During drying	Online/in-line	Fixed RGB or thermal cameras, NIR or selected spectral bands, mass and chamber sensors, with optional dielectric or volatile-fingerprint measurements.	Time-resolved moisture progression, surface condition, heat-load heterogeneity, process state, and quality-risk proxies.	Endpoint estimation, warning generation, operator recommendation, or bounded control; machine vision plus automatic weighing has been demonstrated for jujube drying [129].
After drying	Offline/at-line	Laboratory moisture or water-activity analysis, texture and chemical assays, GC–MS/GC–O or sensory tests, supported by imaging or spectroscopy.	Final stability, texture, nutrient retention, volatile composition, and sensory quality.	Batch verification, root-cause analysis, model updating, and confirmation of proxy meaning [93].
After drying	Online/in-line	Outlet RGB, NIR, or thermal inspection combined with check-weighing and, where justified, volatile or dielectric sensing.	Residual non-uniformity, surface defects, temperature, moisture proxies, and lot-to-lot drift.	Sorting, release or rework decisions, and feedback to upstream dryer settings [131].

## Data Availability

No new data were created or analyzed in this study. Data sharing is not applicable to this article.

## Supplementary Materials

The following supporting information can be downloaded at: https://www.mdpi.com/article/10.3390/foods15173122/s1; Table S1 provides the Web of Science Core Collection search and study-selection framework; Table S2 summarizes operational terminology for sensing, model evaluation, and process deployment.
