# Supplementary material for: Non-Destructive Sensing and Intelligent Quality Prediction During Fruit Drying: From Quality Formation to Decision Support
Source: Foods. 2026 Sep 2;15(17):3122. doi: 10.3390/foods15173122 (PMC13565016; doi:10.3390/foods15173122)
Supplement: Supplementary file 1 [file foods-15-03122-s001.zip › foods-4471323-supplementary.pdf]

Supplementary Materials

Non-Destructive Sensing and Intelligent Quality Prediction During Fruit Drying: From Quality Formation to Decision Support

Kai Zhang [1], Qingqing Yuan [1], Tianrui Liu [1], Roujia Zhang [1], Lilang Li [2], Yu Wang [2], Siyao Liu [3,*] and Chenguang Zhou [1,*]

Table S1. Literature search and study-selection framework.

Item	Specification
**Database and coverage**	Web of Science Core Collection; Topic (TS) field, covering titles, abstracts, author keywords, and Keywords Plus; searched from database inception to 13 August 2026.
**Search development and scope**	The query was developed from the review question and terminology across the initial reference set. It combined fruit and drying terms with non-destructive sensing, intelligent prediction or process-control methods, and moisture or quality outcomes.
**Complete executable query**	TS=(((fruit* OR apple* OR banana* OR mango* OR grape* OR raisin* OR berr* OR jujube OR kiwifruit OR apricot* OR peach* OR papaya* OR pear* OR citrus OR orange* OR plum* OR noni OR goji) NEAR/3 (drying OR dehydration OR dried OR dryer*)) AND (("non-destructive" OR nondestructive OR "non-invasive" OR noninvasive OR spectroscopy OR hyperspectral OR "near infrared" OR "near-infrared" OR NIR OR "machine vision" OR "computer vision" OR "low-field nuclear magnetic resonance" OR "low-field NMR" OR LF-NMR OR "magnetic resonance imaging" OR MRI OR thermography OR "thermal imaging" OR "electronic nose" OR e-nose OR dielectric OR impedance OR ultrasonic OR ultrasound OR sensor* OR imaging OR multimodal OR "sensor fusion" OR "data fusion") OR ("artificial intelligence" OR "machine learning" OR "deep learning" OR prediction OR monitoring OR "process analytical technology" OR "closed-loop" OR control)) AND (quality OR moisture OR "water activity" OR color OR colour OR texture OR firmness OR shrinkage OR structure OR bioactive* OR phenolic* OR flavonoid* OR vitamin* OR aroma OR flavor OR flavour OR volatile* OR endpoint))
**Record identification and selection**	The search returned 4,286 unique records after duplicate batch exports were reconciled by Web of Science accession number. Of these, 4,279 were Articles or Review Articles, 4,180 were in English, and 4,177 remained after three retracted records were removed. Focused title-and-abstract assessment retained 275 records for article-level consideration.
**Focused study selection**	The focused literature linked an active fruit-drying or dehydration process to a named non-destructive sensing or modelling method and to measurement, monitoring, prediction, classification, endpoint detection, control, moisture, or another quality attribute. Studies limited to fresh-fruit grading, storage, dietary effects of dried fruit, wine production, contamination monitoring, unrelated-food formulation, or analytical methods without a fruit-drying question were not retained in this set.
**Contextual literature**	Where fruit-drying studies did not resolve a sensing mechanism or deployment problem, literature from fresh-fruit assessment, other foods, and related production systems was used to interpret transferable principles and engineering constraints in the relevant discussion.
**Synthesis**	The retained literature was synthesized as a critical narrative review. Full texts were checked for study-specific quantitative claims, including sample size, spectral range, validation design, and performance statistics. Heterogeneity in targets, sampling units, preprocessing, validation, and metrics precluded formal meta-analysis.

Table S2. Glossary of terminology used for sensing, artificial intelligence, model evaluation, and process deployment in this review.

Item	Specification
Sensing modalities and measurement concepts	
**Non-destructive sensing**	Acquisition of product or process information without materially damaging the sample. In fruit drying, it includes repeated imaging, spectroscopy, magnetic-resonance, acoustic, electrical, mass, and volatile-sensing measurements.
**Multimodal sensing**	Coordinated acquisition from two or more sensor types that describe complementary aspects of the drying product or environment, such as surface appearance, moisture-related absorption, temperature, mass loss, or volatile change.
**Machine/computer vision and red-green-blue (RGB) imaging**	Camera-based analysis of visible images. RGB systems provide rapid measurements of color, area, shape, shrinkage, wrinkling, and visible defects and are therefore well suited to surface surveillance during drying.
**Three-dimensional (3D) and point-cloud imaging**	Geometric imaging that reconstructs depth, surface relief, or a cloud of spatial coordinates. It extends two-dimensional appearance analysis by quantifying thickness, volume, warping, and non-uniform shrinkage.
**Thermal imaging**	Non-contact measurement of surface-temperature distributions from emitted infrared radiation. During drying, thermal images reveal uneven heat loading and local thermal anomalies rather than internal moisture or chemical composition directly.
**Near-infrared (NIR), visible-near-infrared (Vis-NIR), and Fourier-transform NIR (FT-NIR) spectroscopy**	Spectroscopic measurements dominated by overtone and combination absorptions associated with water and organic constituents. Vis-NIR extends acquisition into the visible range, whereas FT-NIR uses interferometric acquisition to obtain NIR spectra.
**Hyperspectral imaging (HSI)**	Imaging spectroscopy that records a spectrum at each image location, producing a spatial-spectral data cube. It can map surface and near-surface heterogeneity but generates more data than RGB, multispectral, or point spectroscopy.
**Reflectance, transmittance, and transflectance acquisition**	Optical geometries distinguished by how light reaches the detector. Reflectance records light returned from the sample, transmittance records light passing through it, and transflectance records light that traverses the sample and is returned by a reflector.
**Multispectral, selected-band, and short-wave infrared (SWIR) imaging**	Imaging with a limited set of diagnostically chosen wavelength bands. Selected-band systems can use light-emitting diodes or band-pass filters, while SWIR denotes the longer-wavelength infrared region often used for water- and composition-related responses.
**Laser-light backscattering imaging (LLBI) and biospeckle imaging**	Optical methods that characterize light scattering from tissue. LLBI analyzes the spatial backscattering pattern, whereas biospeckle imaging analyzes temporal fluctuations in coherent-light speckle patterns associated with changing tissue properties.
**Low-field nuclear magnetic resonance (LF-NMR) and magnetic resonance imaging (MRI)**	Magnetic-resonance methods sensitive to proton relaxation and water mobility. LF-NMR summarizes water populations within the measured sample volume, whereas MRI spatially resolves internal water distribution and structural heterogeneity.
**Ultrasound sensing**	Use of acoustic propagation through the product to obtain information from velocity, attenuation, reflection, or impedance. These responses reflect density gradients, interfaces, porosity, and structural heterogeneity.
**Dielectric and impedance sensing**	Electrical measurements that respond to water content, ionic mobility, temperature, electrode contact, and sample geometry. Their effective sampling region depends on probe design and field distribution.
**Electronic nose (e-nose), gas-sensor array, and volatile fingerprint**	An array of partially selective gas sensors that records a composite headspace-response pattern. The resulting volatile fingerprint follows changes in odor space but does not identify individual aroma compounds.

Item	Specification
**Electronic tongue, electronic eye, and colorimetric sensor array**	Cross-responsive sensing systems that generate pattern-level descriptors from liquid chemistry or visually encoded chemical reactions. Their value arises from the collective response pattern rather than from a single compound-specific channel.
**Gas chromatography-mass spectrometry (GC-MS) and gas chromatography-olfactometry (GC-O)**	Reference methods used to interpret volatile-sensor outputs. GC-MS separates and identifies volatile compounds, while GC-O links chromatographic peaks to odors perceived by a trained assessor.
**Gravimetric and mass sensing**	Measurement of product mass or mass loss during drying. It provides a bulk dehydration trajectory that can support moisture-ratio and endpoint estimation when initial mass, dry mass, and sampling conditions are defined.
**Process and environmental sensors**	Measurements of the drying environment and equipment state, including temperature, relative humidity, airflow, pressure, energy use, and residence time. These variables provide the process context for interpreting product-sensor signals.
Artificial intelligence, chemometrics, and data fusion	
**Artificial intelligence (AI)**	The broad family of computational methods used for perception, prediction, optimization, and decision support. In sensor-guided drying, AI links measurements to product-state estimates or process actions.
**Machine learning (ML) and deep learning (DL)**	ML learns relationships from data rather than relying only on fixed equations. DL is a subset of ML based on multilayer neural networks that can learn hierarchical features from spectra, images, or time series.
**Chemometrics and calibration model**	Chemometrics applies multivariate statistics and computation to chemical or spectral measurements. A calibration model relates sensor variables to reference values or classes so that an unmeasured attribute can be estimated from new observations.
**Regression and classification**	Regression predicts a continuous response such as moisture content or a color coordinate, whereas classification assigns an observation to a discrete group such as defect class or browning-severity category.
**Multiple linear regression (MLR), partial least squares (PLS/PLSR), and PLS discriminant analysis (PLS-DA)**	MLR models a response as a linear combination of predictors. PLS regression constructs latent variables that jointly represent predictors and response, while PLS-DA adapts the latent-variable formulation to categorical discrimination.
**Support vector machine/regression (SVM/SVR) and Gaussian process regression (GPR)**	SVM separates classes and SVR fits continuous responses using margin-based learning and kernels. GPR provides a probabilistic regression function in which covariance structure represents similarity among observations.
**Random forest/regression (RF/RFR), extreme gradient boosting (XGBoost), and ensemble learning**	Tree-based ensembles combine multiple learners to improve prediction stability. Random forests average decorrelated trees, XGBoost builds boosted trees sequentially, and ensemble learning more broadly combines predictions from several models.
**Artificial neural network (ANN), multilayer perceptron (MLP), convolutional neural network (CNN), and long short-term memory (LSTM)**	ANN is the general neural-network family; an MLP uses fully connected layers, a CNN learns local spatial or spectral patterns, and an LSTM represents temporal dependence through gated recurrent units.
**Multi-output regression and multi-task learning**	Modeling strategies that estimate several quality attributes together. Shared representations can capture relationships among outputs while retaining separate predictions for moisture, color, texture, composition, or flavor-related targets.
**Attention model and graph-based model**	Attention assigns context-dependent weights to informative inputs or regions. Graph-based models represent variables, spatial regions, or modalities as connected nodes so that relationships among them contribute to prediction or fusion.
**Spectral preprocessing: Savitzky-Golay (SG) and standard normal variate (SNV)**	SG filtering smooths spectra or estimates derivatives through local polynomial fitting. SNV centers and scales each spectrum to reduce multiplicative scattering and baseline variation.
**Feature extraction and feature engineering**	Transformation of raw measurements into descriptors used by a model, such as color indices, image texture, morphology, thermal statistics, selected spectral responses, water-state variables, or volatile-pattern features.

Item	Specification
**Feature selection: successive projections algorithm (SPA) and competitive adaptive reweighted sampling (CARS)**	Feature-selection methods that retain informative variables while reducing redundancy. SPA limits collinearity through sequential projection, whereas CARS repeatedly samples and reweights variables to identify a compact predictive subset.
**Region of interest (ROI) and image segmentation**	An ROI is the part of an image selected for analysis. Segmentation separates product tissue from the background or divides it into meaningful regions before color, shape, texture, thermal, or spectral features are calculated.
**Multimodal or data fusion**	Combination of information from different sensors, feature spaces, or models. In drying applications, fusion is most informative when the inputs describe complementary product or process phenomena.
**Data-, feature-, decision-, and model-level fusion**	Data-level fusion combines aligned raw measurements; feature-level fusion joins derived descriptors; decision-level fusion combines separate model outputs; and model-level fusion learns shared or interacting representations within a joint architecture.
**Data alignment and synchronization**	Matching measurements so that different sensors refer to compatible samples, spatial regions, and process times. Alignment provides the common observation unit needed for multimodal modeling.
Model evaluation, transfer, and explanation	
**Calibration/training, validation, and prediction/test sets**	The calibration or training set fits the model, the validation set supports model selection or tuning, and the prediction or test set estimates performance on observations not used during model development.
**Cross-validation (CV), k-fold CV, and leave-one-out CV**	CV repeatedly divides development data into fitting and held-out subsets. k-fold CV rotates among several folds, whereas leave-one-out CV uses one observation as the held-out subset in each iteration.
**Kennard-Stone and sample-set partitioning based on joint *x–y* distances (SPXY)**	Deterministic sample-partition methods. Kennard-Stone spreads selected samples across predictor space, while SPXY considers distances in both predictor and response spaces when forming calibration and prediction subsets.
**External validation**	Evaluation on genuinely independent samples, batches, origins, seasons, instruments, dryers, or operating runs. It examines whether the learned relationship persists beyond the experiment from which the calibration data were obtained.
**Data leakage**	Transfer of information from the held-out data into model fitting or tuning. In drying studies, leakage can arise when repeated observations from the same fruit, slice, batch, or run are divided across development and test subsets.
**Generalization, model robustness, and transferability**	Generalization describes performance on new observations, robustness describes stability under relevant variation, and transferability describes the extent to which a model retains meaning and performance across products, batches, instruments, or dryers.
**Uncertainty estimation**	Quantification of the confidence or dispersion associated with a prediction. It complements a point estimate by indicating when data support a narrow estimate and when the predicted state remains ambiguous.
**Calibration transfer**	Adjustment of spectra, model coefficients, or predictions so that a calibration developed on one instrument can be used with another instrument or a changed hardware configuration.
**Domain adaptation and transfer learning**	Domain adaptation reduces differences between source and target data distributions, whereas transfer learning reuses knowledge from a previously trained model when developing a model for a related product, instrument, or process.
**Domain shift and out-of-distribution (OOD) detection**	Domain shift is a change in the data distribution caused by factors such as cultivar, season, dryer, instrument, or acquisition conditions. OOD detection identifies observations that fall outside the range represented during model development.
**Sensor drift**	A gradual change in sensor response over time that is not caused by the product itself. Fouling, aging, temperature, humidity, illumination, and component replacement can all contribute to drift.

Item	Specification
**Confounding feature and shortcut learning**	A confounding feature varies with both the target and another experimental factor. Shortcut learning occurs when a model exploits an easy correlate, such as elapsed time, tray position, background, or illumination, instead of the intended product signal.
**Explainable artificial intelligence (XAI)**	Methods that describe how model inputs, features, or regions contribute to predictions. Their output characterizes model behavior and can be compared with physically or chemically plausible drying responses.
**Shapley additive explanations (SHAP)**	A game-theoretic attribution method that assigns each feature a contribution to an individual prediction and can aggregate these contributions to summarize broader model behavior.
**Local interpretable model-agnostic explanations (LIME)**	A local surrogate approach that approximates the behavior of a complex model around one selected observation using a simpler interpretable model.
**Gradient-weighted class activation mapping (Grad-CAM)**	A gradient-based visualization that highlights image regions contributing strongly to a convolutional-network output, thereby showing where the model found influential spatial information.
**Masking test**	A diagnostic experiment in which selected wavelengths, image regions, or contextual features are removed or obscured and the resulting change in prediction is observed.
**Performance statistics: R, R^2, R^2_{P}, RMSE, RMSEP, MAE, and mean relative error**	R is the correlation coefficient; R^2 is the coefficient of determination; R^2_{P} refers to a prediction set; RMSE and RMSEP summarize root-mean-square error; MAE is mean absolute error; and mean relative error expresses error relative to the reference value.
Process deployment and control	
**Offline measurement**	Sampling and analysis carried out away from the operating dryer, commonly with a laboratory reference method used to establish moisture, texture, chemical, or sensory values.
**At-line analysis**	Rapid measurement of a removed sample near the dryer. The result remains temporally close to production although the sensor is not integrated into the product stream.
**Online sensing and real-time monitoring**	Automated measurement acquired during operation without routine sample removal. Real-time monitoring denotes acquisition and processing fast enough to follow the process at the intended decision interval.
**In-line sensing**	Measurement by a sensor integrated into the dryer or product flow, with the material observed while it remains within the process.
**Process analytical technology (PAT)**	A process framework that connects timely measurements with interpretation and intervention, allowing product and process states to guide operation rather than relying only on final-product testing.
**Direct measurement and calibrated proxy**	A direct measurement represents the stated physical quantity at the sensor's sampling scale. A calibrated proxy estimates another attribute through an empirical or mechanistic relationship with the measured signal.
**Soft sensor and state estimation**	A soft sensor is a computational estimator that combines available measurements to infer a difficult-to-measure state. State estimation updates the current product or process condition from those measurements and a model.
**Endpoint prediction, decision support, and quality-risk warning**	Endpoint prediction estimates when a drying target will be reached. Decision support translates current and forecast states into an operator-facing recommendation, while a quality-risk warning indicates emerging deviation from an acceptable product trajectory.
**Edge processing, windowed aggregation, adaptive sampling, and event-triggered updating**	Edge processing performs computation near the sensor or dryer. Windowed aggregation summarizes recent observations, adaptive sampling changes acquisition frequency with process state, and event-triggered updating responds to a detected change or risk.
**Fault detection, model updating, and model maintenance**	Fault detection separates abnormal sensor or system behavior from expected product change. Model updating incorporates new data, while model maintenance includes drift checks, recalibration, transfer, documentation, and periodic performance review.

Item	Specification
**Multi-objective optimization, genetic algorithm (GA), and Bayesian optimization**	Multi-objective optimization examines trade-offs among outcomes such as time, energy, moisture stability, color, texture, and nutrient retention. GA searches through evolutionary operations, whereas Bayesian optimization uses a probabilistic surrogate to select informative operating conditions.
**Digital twin, physics-informed neural network (PINN), and hybrid model**	A digital twin is a dynamically updated computational representation of the drying system. PINNs incorporate governing equations or physical constraints into neural-network learning, while hybrid models combine mechanistic and data-driven components.
**Model-predictive control (MPC)**	A receding-horizon control strategy that predicts future process behavior, optimizes a sequence of control moves under constraints, applies the first move, and repeats the calculation after new measurements arrive.
**Closed-loop control and bounded actuation**	Closed-loop control returns sensor-derived state estimates to the controller so that operating conditions can be adjusted automatically. Bounded actuation limits each adjustment to a predefined safe range.
**Latency, control interval, fail-safe action, manual override, and post-drying verification**	Latency is the delay from sensing to an available decision, and the control interval is the time between possible interventions. Fail-safe action, manual override, and post-drying verification provide complementary safeguards when automated estimates or adjustments are uncertain.
